# DUSP26 Regulated by YY1 Lactylation Couples Osteogenesis and Angiogenesis by Dephosphorylating PMPCB at Y141 and Activating Mitophagy in BMSCs

**DOI:** 10.1002/advs.78514

**Published:** 2026-10-28

**Authors:** Yunpeng Lv, Feng Wu, Xiongjie Liang, Chong Liu, Wenhui Guo, Kejian Zhang, Jiawen Niu, Wenjie Jiang, Hongjian Xu, Anlong Jiang, Yang Zheng, Enming Yu, Xintao Wang, Jinglong Yan, Jingjun Xia, Chengchao Song

**Affiliations:** ^1^ Department of Orthopedic Surgery The Second Affiliated Hospital of Harbin Medical University Harbin China; ^2^ Department of Ultrasound The Second Affiliated Hospital of Harbin Medical University Harbin China; ^3^ Department of Cardiology The Second Affiliated Hospital of Harbin Medical University Harbin China

**Keywords:** BMSCs, DUSP26, mitophagy PMPCB, YY1

## Abstract

Bone marrow mesenchymal stem cells (BMSCs) support bone formation through osteogenic differentiation and pro‐angiogenic paracrine activity, but the mechanisms coordinating these functions remain incompletely understood. Here, we identified dual‐specificity phosphatase 26 (DUSP26) as a regulator of BMSC osteogenesis and mitochondrial homeostasis. DUSP26 expression increased during osteogenic differentiation and was reduced in osteoporotic femoral tissues. p300 gain‐ and loss‐of‐function experiments, together with analysis of the Yin Yang1(YY1) K183R mutant, supported the conclusion that p300‐regulated YY1 lactylation contributes to DUSP26 transcriptional activation. DUSP26 interacted with the mitochondrial processing peptidase subunit beta(PMPCB) and reduced its tyrosine phosphorylation, with mass spectrometry and mutational analyses implicating Y141 as involved in this regulation. Functional assays supported a model in which DUSP26 limits mitochondrial processing peptidase (MPP)‐mediated PTEN‐induced kinase 1 (PINK1) processing and promotes PINK1‐dependent mitophagy, thereby supporting mitochondrial homeostasis. DUSP26 enhanced osteogenic differentiation and the pro‐angiogenic activity of BMSC‐conditioned medium(CM), whereas its knockdown impaired bone formation and reduced type H vessels in vivo. Furthermore, BMSC‐derived exosomes carrying a runt‐related transcription factor 2 (RUNX2) promoter‐driven DUSP26 plasmid produced preferential transgene expression in osteogenic‐lineage cells and attenuated ovariectomy‐induced bone loss in mice. These findings link lactylation‐associated transcriptional regulation to mitochondrial protein processing and support further preclinical evaluation of DUSP26‐directed approaches for osteoporosis.

## Graphical abstract

p300‐associated YY1 lactylation promotes DUSP26 expression in BMSCs. By regulating PMPCB phosphorylation and PINK1 processing, DUSP26 supports mitophagy, osteogenic differentiation, and pro‐angiogenic factor secretion. BMSC‐derived exosomes carrying a RUNX2 promoter‐driven DUSP26 plasmid favor osteogenic‐lineage transgene expression, partially attenuate bone loss, and increase type H vessel abundance in ovariectomized mice.

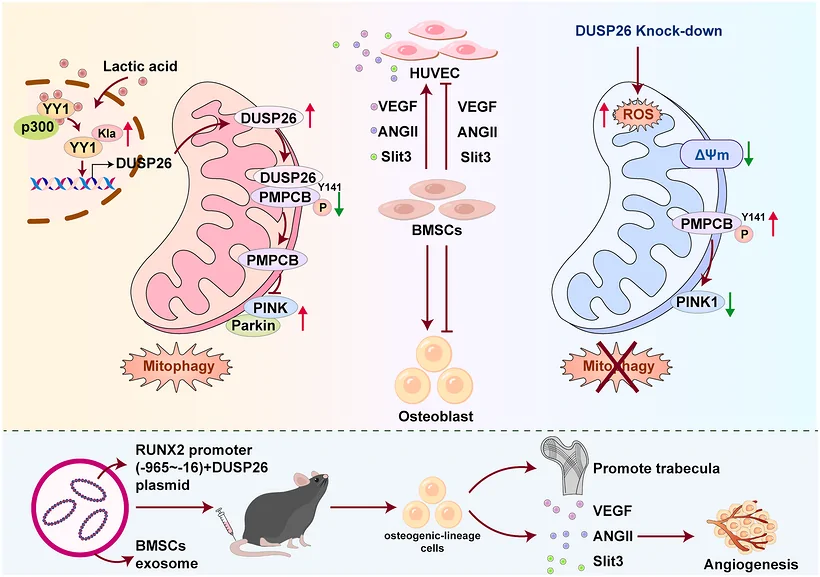

## 1 Introduction

With the ongoing shift in global population structure, the aging population is increasing steadily, contributing to the rising incidence of osteoporosis [1]. Osteoporosis is a systemic skeletal disorder characterized by reduced bone mass and microarchitectural deterioration [2]. Its pathological features include oxidative stress, chronic inflammation, and impaired bone‐vascular coupling, among which disruption of bone‐vascular coupling is considered an important contributor to the development and progression of osteoporosis [3, 4, 5]. Specifically, the differentiation of BMSCs is markedly impaired, accompanied by a loss of their pro‐angiogenic capacity. In addition, the reduced angiogenic activity of endothelial cells and the altered secretory profile of dysfunctional BMSCs contribute to bone loss and destruction of bone microarchitecture [6]. However, the key regulatory factors governing bone‐vascular coupling remain poorly defined.

Mitochondrial function is important for osteogenic differentiation. In diabetic models, inhibition of PINK1/dynamin‐related protein 1(Drp1)‐mediated mitophagy has been linked to impaired osteoblastogenesis [7]. Mitophagy selectively removes damaged or dysfunctional mitochondria and contributes to mitochondrial quality control and the regulation of oxidative stress [8]. In BMSCs exposed to oxidative stress, mitophagy has been shown to support cell survival [9]. Mitochondrial transfer can also enhance BMSC osteogenic differentiation and bone defect repair, accompanied by increased oxidative phosphorylation and adenosine triphosphate(ATP) production [10]. Metabolic dysfunction and oxidative stress are implicated in diabetic osteoporosis [11], while age‐dependent reactive oxygen species (ROS)‐related signaling can influence osteogenesis‐angiogenesis coupling through hypoxia‐inducible factor 1 alpha(HIF1α) [12]. These findings suggest that mitochondrial homeostasis may affect both the osteogenic function of BMSCs and their interactions with the vascular microenvironment. Autophagy is involved in the regulation of bone metabolism [13], and collagen type VI alpha 3 chain(COL6A3) overexpression has been reported to improve BMSC differentiation under inflammatory conditions in association with enhanced mitophagy [14]. Mitophagy has therefore been proposed as a potential target in age‐related bone diseases [15]. However, the molecular regulators that connect mitochondrial quality control to both osteogenic differentiation and pro‐angiogenic paracrine activity in BMSCs remain incompletely defined.

Dual‐specificity phosphatases (DUSPs) regulate intracellular signaling and influence a broad range of physiological processes [16]. Members of the DUSP family have also been implicated in mitophagy and osteogenic differentiation. For example, dual‐specificity phosphatase 12 interacts with heat shock protein beta‐8 to promote mitophagy and alleviate myocardial ischemia‐reperfusion injury [17]. Dual‐specificity phosphatase 5 promotes osteogenic differentiation of mesenchymal stromal cells by activating SMAD Family Member 1 signaling through a small C‐terminal phosphatases 1 and 2‐dependent mechanism [18]. DUSP26 is another member of this family. Loss of DUSP26 in neurons increases mitochondrial ROS production and activates p38 signaling and inflammatory responses [19]. In a different tissue context, DUSP26 promotes calcification in human valvular interstitial cells by antagonizing mouse double minute 2 homolog‐mediated dipeptidyl peptidase 4 ubiquitination [20]. These findings indicate that DUSP26 can regulate mitochondrial function and calcification, but its role in BMSC osteogenic differentiation and bone‐vascular coupling remains unclear.

Here, we investigated whether DUSP26 coordinates osteogenic differentiation and pro‐angiogenic paracrine activity in BMSCs through mitochondrial quality control. Public databases were used to nominate candidate upstream regulators and interacting proteins for experimental validation. We examined p300‐associated YY1 lactylation as an upstream regulatory mechanism and assessed how DUSP26‐dependent regulation of PMPCB phosphorylation influences PINK1 processing and mitophagy. We further evaluated BMSC‐derived exosomes carrying a RUNX2 promoter‐driven DUSP26 plasmid as a strategy to promote preferential transgene expression in osteogenic‐lineage cells and attenuate bone loss in an ovariectomy model. These findings provide a potential biological target and a precision therapeutic strategy for osteoporosis.

## 2 Results

### 2.1 DUSP26 Plays a Critical Role in the Osteogenic Differentiation of BMSCs

To investigate the relationship between DUSP26 and osteoporosis, we first examined DUSP26 expression in femoral tissues from osteoporotic mice, which exhibited sparse and thinned trabeculae (Figure 1A). Immunofluorescence(IF) staining and Western blotting showed that DUSP26 protein expression was significantly lower in osteoporotic femoral tissues than in control tissues (Figure 1B,C). We next assessed the dynamic expression pattern of DUSP26 during the osteogenic differentiation of BMSCs. As the messenger ribonucleic acid (mRNA) and protein levels of alkaline phosphatase (ALP) and RUNX2 progressively increased, DUSP26 expression displayed a similar upward trend (Figure 1D,E). In addition, IF analysis demonstrated that DUSP26 staining intensity was markedly increased after 7 days of osteogenic induction in BMSCs (Figure 1F).

**FIGURE 1:**
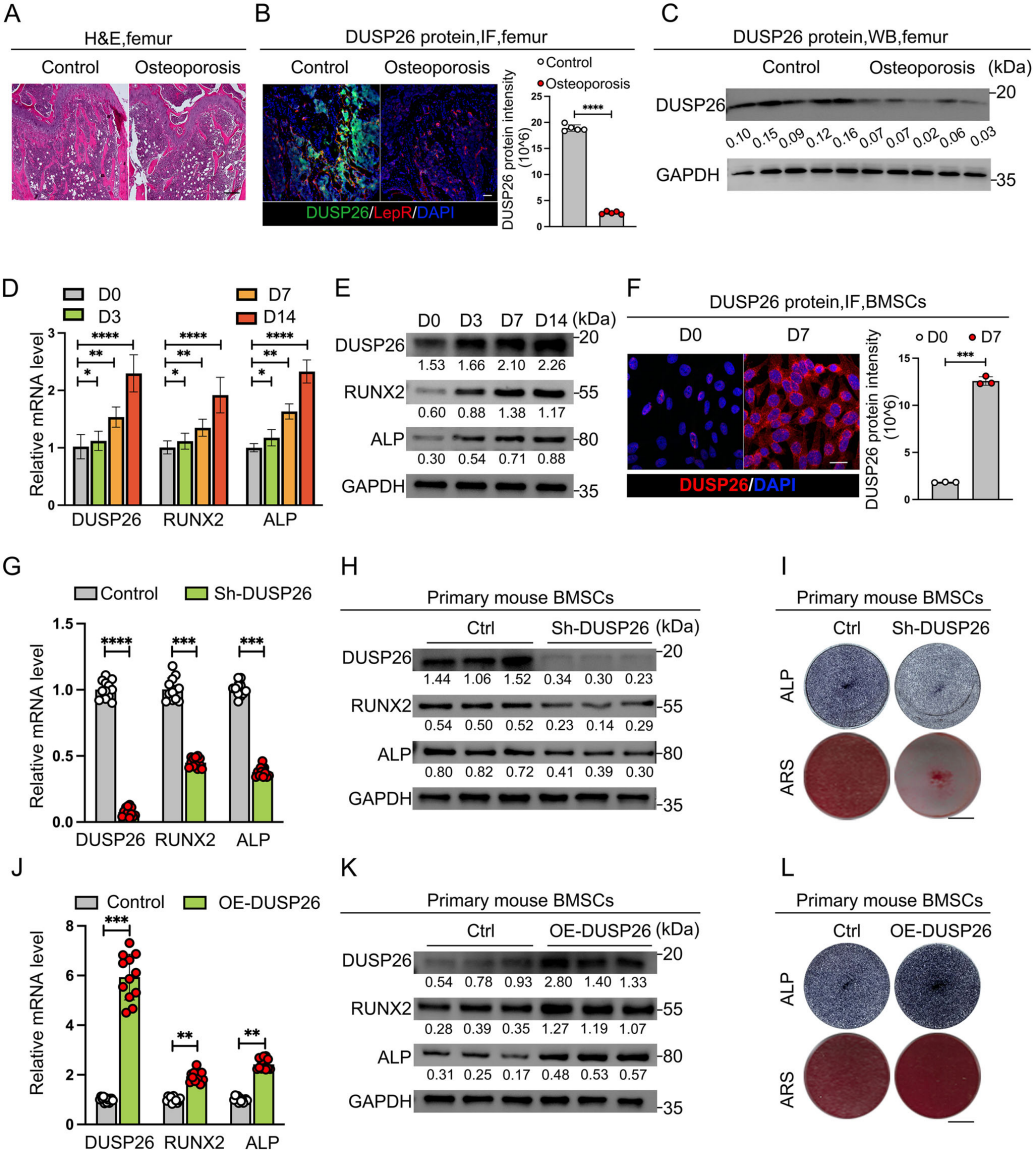
DUSP26 mediates a positive regulatory role in bone formation. (A–C) Hematoxylin and eosin (H&E), IF staining, and Western blotting were performed on femurs from healthy C57BL/6J mice (Control, *n* = 5) and mice diagnosed with osteoporosis according to pathological examination (Osteoporosis, *n* = 5). The staining levels of DUSP26 were quantified using ImageJ software. (A) Scale bar = 200 µm; (B) Scale bar = 100 µm. (D–F) reverse transcription quantitative polymerase chain reaction (RT‐qPCR) (*n* = 12), Western blotting (*n* = 3), and IF staining (*n* = 3) demonstrated the expression changes of DUSP26 in BMSCs after osteogenic induction. (F) Scale bar = 20 µm. (G–I) RT‐qPCR (*n* = 12), Western blotting (*n* = 3), ALP, and ARS staining (*n* = 3) were used to evaluate the impact of DUSP26 knockdown on the osteogenic differentiation of BMSCs. (I) Scale bar = 5 mm. (J–L) RT‐qPCR (*n* = 12), Western blotting (*n* = 3), ALP, and ARS staining (*n* = 3) were used to evaluate the impact of DUSP26 overexpression on the osteogenic differentiation of BMSCs. (L) Scale bar = 5 mm. ^**^ *p* < 0.01, ^***^ *p* < 0.001,^****^ *p* < 0.0001, ns, not significant.

To further determine the function of DUSP26 in osteogenesis, DUSP26 was knocked down or overexpressed by plasmid transfection. Compared with the vector group, knockdown of DUSP26 significantly decreased the mRNA and protein levels of ALP and RUNX2, accompanied by reduced ALP and alizarin red s (ARS) staining intensity (Figure 1G–I). In contrast, overexpression of DUSP26 increased the mRNA and protein levels of ALP and RUNX2 and enhanced both ALP and ARS staining (Figure 1J–L). Collectively, these findings indicate that DUSP26 is essential for the osteogenic differentiation of BMSCs and may be involved in the pathogenesis of osteoporosis.

### 2.2 DUSP26 Promotes Angiogenesis in human umbilical vein endothelial cells(HUVECs) by Regulating the Paracrine Function of BMSCs

Our previous studies demonstrated that BMSCs can promote the formation of type H vessels through exosome‐mediated mechanisms [4]. To determine whether DUSP26 alters the paracrine pro‐angiogenic activity of BMSCs, we measured the levels of vascular endothelial growth factor (VEGF), angiopoietin‐2 (ANG2), and slit guidance ligand 3 (SLIT3) in BMSC culture supernatants. DUSP26 knockdown significantly decreased the concentrations of VEGF, ANG2, and SLIT3 in the culture medium of BMSCs (Figure S1A). Conversely, overexpression of DUSP26 increased the levels of these pro‐angiogenic factors (Figure S1B). To further determine whether DUSP26 influences angiogenesis through the paracrine activity of BMSCs, we collected BMSC supernatants to prepare CM for HUVEC culture (Figure 2A). Compared with Dulbecco’s modified Eagle’s medium/Ham’s F‐12 (DMEM/F12) alone or CM derived from control BMSCs, CM from DUSP26‐overexpressing BMSCs significantly increased the number of 5‐ethynyl‐2′‐deoxyuridine (EdU)‐positive HUVECs (Figure 2B). Similarly, the cell counting kit‐8 (CCK‐8) assay showed that CM from DUSP26‐overexpressing BMSCs significantly enhanced HUVEC proliferation (Figure 2C). Transwell assays further showed that CM derived from DUSP26‐overexpressing BMSCs significantly increased the number of migrated HUVECs (Figure 2D). In scratch assays, the wound closure area of HUVECs was markedly greater at 6 and 12 h in the CM from DUSP26‐overexpressing BMSCs group than in the DMEM/F12 or control CM groups (Figure 2E). Tube formation assays showed that CM from DUSP26‐overexpressing BMSCs significantly increased the number of nodes formed by HUVECs (Figure 2F). These findings indicate that DUSP26 enhances the pro‐angiogenic effects of BMSC‐CM on HUVECs.

**FIGURE 2:**
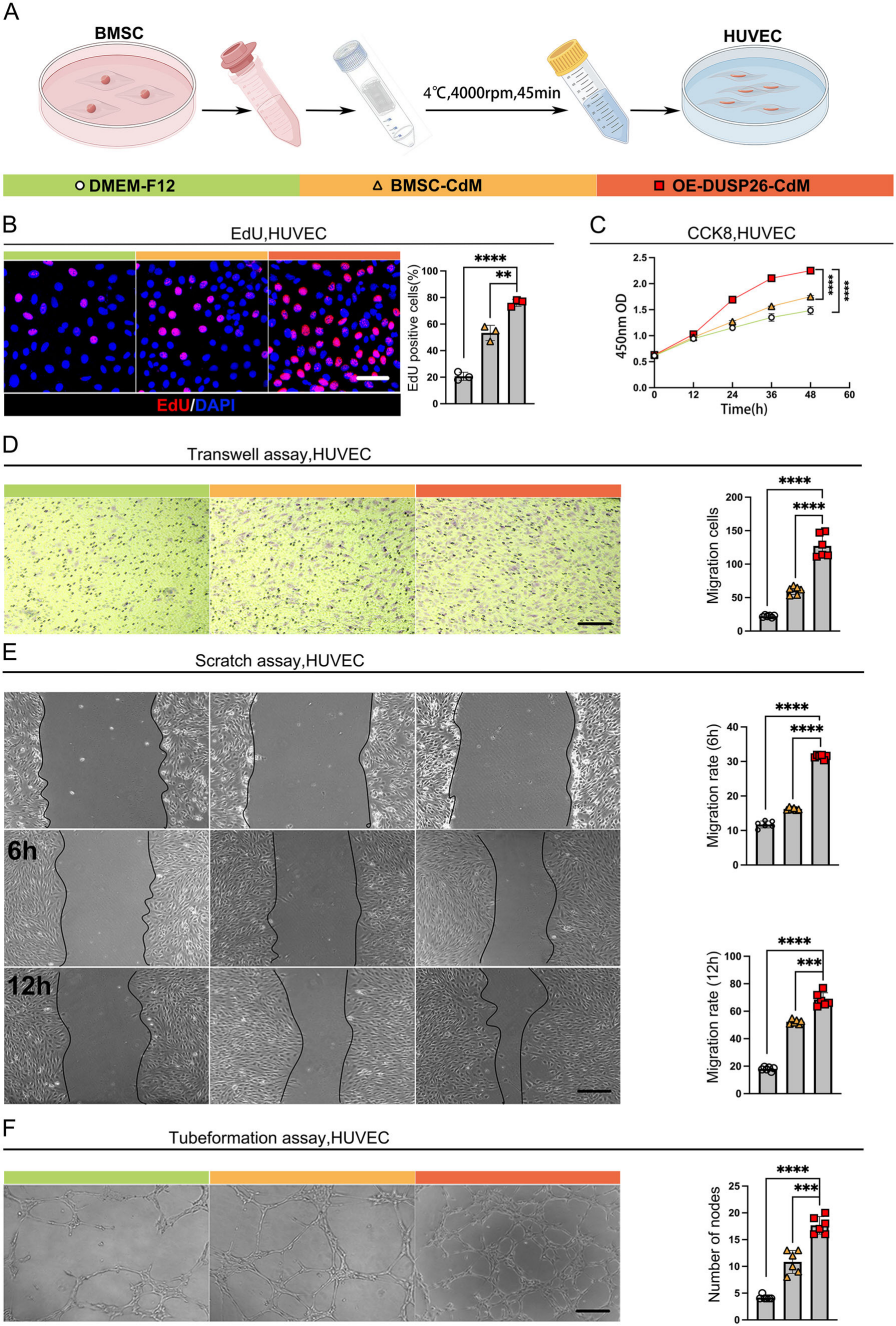
DUSP26 enhances the paracrine pro‐angiogenic activity of BMSCs in HUVEC assays. (A) Schematic diagram of CM preparation. (B, C) EdU (*n* = 3) and CCK‐8 assays (*n* = 12) were performed to evaluate the effects of different culture medium conditions on the proliferation capacity of HUVECs. (B) Scale bar = 50 µm. (D–F) Transwell, scratch, and tube formation assays were conducted to assess the invasion, migration, and number of network nodes of HUVECs under various culture medium conditions (*n* = 3). Scale bar = 100 µm.

### 2.3 DUSP26 Regulates Bone Formation and Type H Vessel Formation In Vivo

To evaluate the role of DUSP26 in vivo, adeno‐associated virus (AAV) encoding short hairpin RNA targeting DUSP26 (shDUSP26) was injected intramedullarily into mice to suppress DUSP26 expression. H&E staining showed that DUSP26 knockdown disrupted bone architecture, as evidenced by sparse trabeculae and reduced cortical thickness (Figure 3A). Consistently, micro‐computed tomography (Micro‐CT) analysis demonstrated that DUSP26 knockdown impaired bone microarchitecture, with decreases in bone mineral density (BMD), bone volume/tissue volume (BV/TV), trabecular thickness (Tb.Th), trabecular number (Tb.N), and cortical thickness (Cort.Th), as well as an increase in trabecular separation (Tb.Sp) (Figure 3B).

**FIGURE 3:**
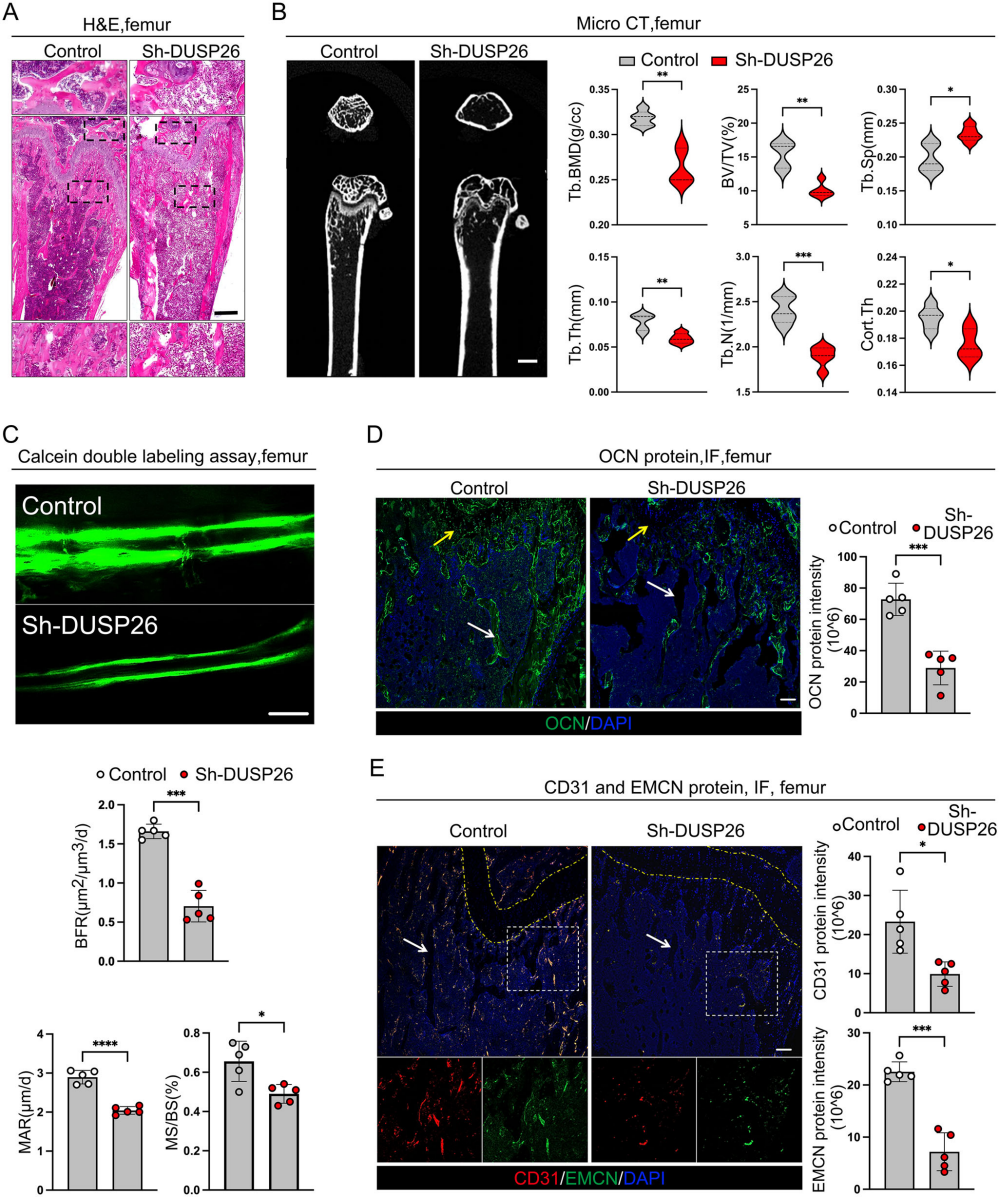
Changes in mouse bones after DUSP26 knockdown. (A, B) H&E and Micro‐CT imaging for evaluating bone changes after DUSP26 knockdown (*n* = 5). (A) Scale bar = 200 µm. (B) Scale bar = 500 µm. (C) Representative images of calcein labeling and quantitative analysis of MAR, MS/BS, and BFR in femurs of DUSP26 knockdown and control mice (*n* = 5). Scale bar = 100 µm. (D, E) Immunofluorescence (IF) staining for assessing expression of osteocalcin (OCN), cluster of differentiation 31 (CD31), and endomucin (EMCN) proteins in distal femoral bones of DUSP26 knockdown mice (*n* = 5). Scale bar = 100 µm. The yellow arrow refers to the growth plate, while the white arrow refers to the trabeculae.^**^ *p* < 0.01, ^***^ *p* < 0.001,^****^ *p* < 0.0001, ns, not significant.

Calcein double‐labeling assays further showed that inhibition of DUSP26 reduced the bone formation rate (BFR), mineral apposition rate (MAR), and mineralizing surface/bone surface (MS/BS) (Figure 3C). IF staining revealed that the fluorescence intensity of the osteoblast‐related protein OCN was significantly decreased in bone tissues following shDUSP26 injection (Figure 3D). In addition, the density of CD31‐ and EMCN‐double‐positive type H vessels was markedly reduced in bone tissues from shDUSP26‐treated mice (Figure 3E). Together, these results indicate that DUSP26 plays a critical role in bone formation and type H vessel formation in vivo.

### 2.4 p300 Enhances DUSP26 Expression Through YY1 Lactylation

To investigate the molecular mechanism underlying the upregulation of DUSP26 during osteogenic differentiation, we analyzed the promoter region of the mouse DUSP26 gene using public databases. Potential transcription factors binding to the DUSP26 promoter were predicted using AnimalTFDB, Cistrome DB, and PROMO. Among the candidate factors, YY1 was identified as the only transcription factor predicted by all three databases to bind the DUSP26 promoter (Figure 4A). The JASPAR database further predicted a YY1‐binding site within the promoter region of DUSP26 (Figure 4B). Chromatin immunoprecipitation(ChIP)‐qPCR confirmed that YY1 bound to the region located from +213 to +220 bp relative to the transcription start site of DUSP26 (Figure 4C). In addition, dual‐luciferase reporter assays showed that co‐transfection of YY1 with the pGL3‐DUSP26‐wild type (WT) reporter significantly increased luciferase activity during osteogenic differentiation, whereas co‐transfection with the mutant promoter reporter pGL3‐DUSP26‐MUT had no significant effect (Figure 4D). Formaldehyde‐assisted isolation of regulatory elements coupled with qPCR (FAIRE‐qPCR) further showed that YY1 knockdown markedly reduced chromatin accessibility at the DUSP26 promoter region (Figure S2A). These findings indicate that YY1 directly activates DUSP26 transcription.

**FIGURE 4:**
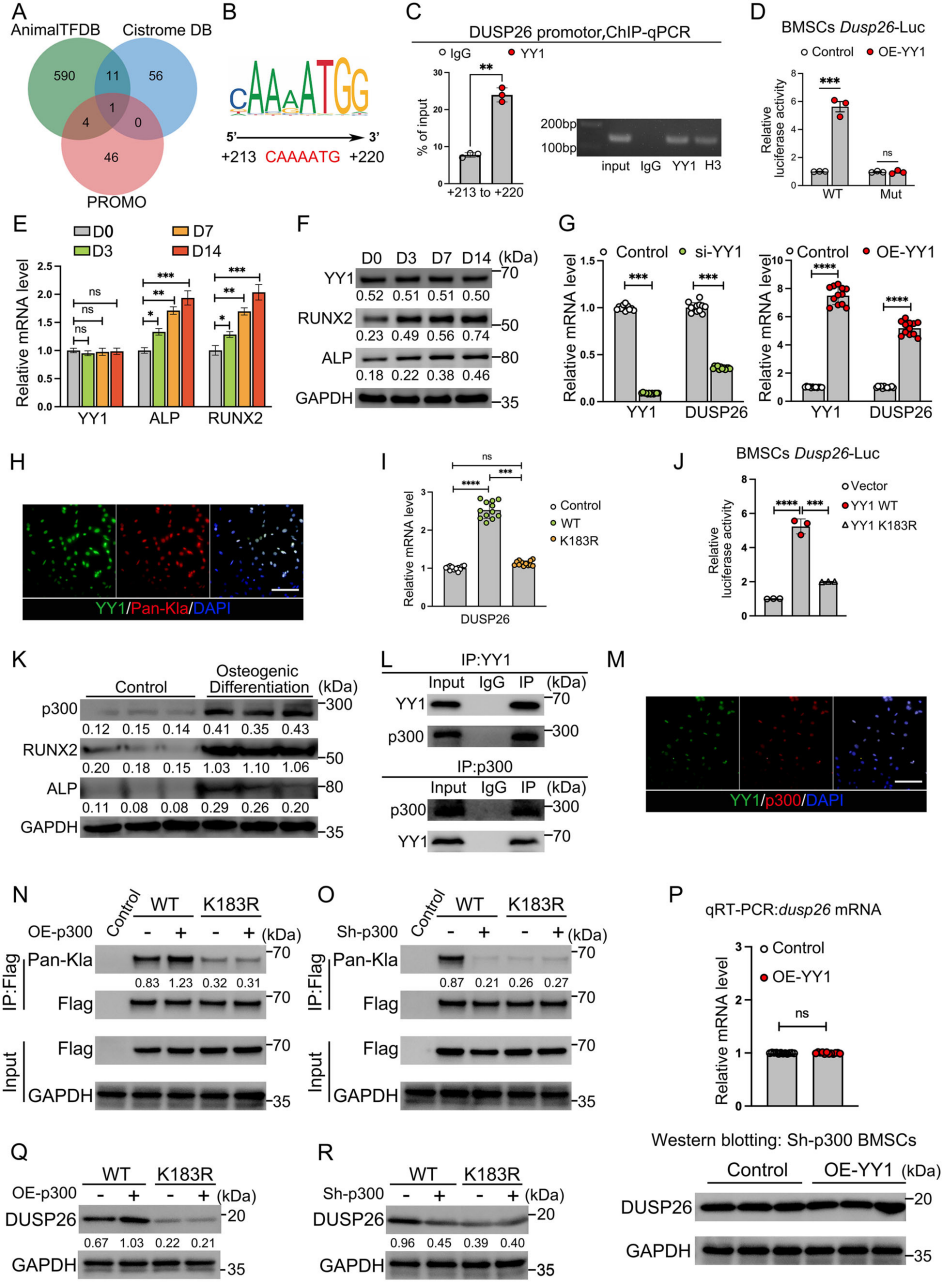
Mechanism by which YY1 promotes DUSP26 mRNA transcription. (A) Venn diagram identifying YY1 as a candidate transcription factor. (B) Potential YY1 binding sites of the DUSP26 promoter. (C, D) ChIP‐qPCR and Dual‐luciferase reporter assay were employed to evaluate the binding of YY1 to the DUSP26 promoter and its transcriptional activity. (*n* = 3). (E, F) RT‐qPCR (*n* = 12) and Western blotting (*n* = 3) were employed to evaluate the expression changes of YY1 after osteogenic induction of BMSCs. (G) RT‐qPCR analysis of YY1's effect on DUSP26 mRNA expression following osteogenic induction (*n* = 12). (H) IF staining to demonstrate the co‐localization of YY1 and pan‐lysine lactylation (pan‐Kla) (*n* = 3). Scale bar = 50 µm. (I) RT‐qPCR (*n* = 12) was employed to detect the lactylation levels of K183R and the impact of YY1 lactylation on DUSP26 mRNA expression. (J) A dual‐luciferase reporter assay was used to assess the relative transcriptional activity of the DUSP26 promoter in BMSCs transfected with empty vector, YY1 WT, or YY1 K183R. (*n* = 3). (K) Western blotting was used to detect the expression of p300 after osteogenic induction of BMSCs. (*n* = 3). (L, M) Co‐immunoprecipitation(Co‐IP) and IF staining were performed to detect the interaction between YY1 and p300 protein. (*n* = 3). (M) Scale bar = 50 µm. (N, O) Western blotting was used to assess the effects of p300 on the lactylation levels of YY1 WT and the YY1 K183R. (*n* = 3). (P) RT‐qPCR (*n* = 12) and Western blotting (*n* = 3) were used to assess the effects of YY1 overexpression on DUSP26 expression in p300‐knockdown BMSCs. (Q, R) Western blotting was used to assess the effects of p300 on DUSP26 expression in BMSCs transfected with YY1 WT or YY1 K183R (*n* = 3).^**^ *p* < 0.01, ^***^ *p* < 0.001,^****^ *p* < 0.0001, ns, not significant.

We next investigated how YY1 promotes DUSP26 transcription during osteogenic differentiation. RT‐qPCR and Western blotting showed that YY1 expression did not change significantly during osteogenic differentiation of BMSCs (Figure 4E,F). However, YY1 knockdown by shRNA decreased DUSP26 mRNA expression under osteogenic conditions, whereas YY1 overexpression significantly increased DUSP26 mRNA levels (Figure 4G). Notably, under non‐osteogenic conditions, neither YY1 overexpression nor YY1 knockdown significantly altered DUSP26 mRNA expression, suggesting that YY1‐dependent transcriptional activation of DUSP26 is associated with the osteogenic state (Figure S2B).

Lysine lactylation is a recently identified post‐translational modification, and YY1 has been reported to undergo lactylation at K183 [21]. IF analysis showed substantial co‐localization between YY1 and pan‐Kla signals (Figure 4H). Immunoprecipitation assays further demonstrated that YY1 lactylation was significantly increased during osteogenic differentiation of BMSCs (Figure S2C).

To determine whether YY1‐mediated transcriptional activation of DUSP26 depends on K183, we generated a Flag‐tagged YY1‐K183R in which lysine 183 was replaced with arginine, thereby preventing lactylation at this position. Under osteogenic conditions, YY1‐K183R exhibited markedly lower lactylation than YY1‐WT (Figure S2D). Consistently, further analysis showed that DUSP26 mRNA levels were significantly lower in the YY1‐K183R group than in the YY1‐WT group and were comparable to those in the control group (Figure 4I). Dual‐luciferase reporter assays showed that YY1‐WT significantly enhanced DUSP26 promoter‐driven luciferase activity, whereas YY1‐K183R exhibited a significantly reduced ability to enhance this activity (Figure 4J). These findings indicate that K183 is important for YY1‐mediated activation of the DUSP26 promoter and that YY1 contributes to maintaining an accessible chromatin state at this regulatory region.

We next investigated the involvement of p300 in this process. Western blotting showed that p300 protein expression was significantly increased during osteogenic differentiation of BMSCs (Figure 4K). Co‐IP assays demonstrated an interaction between p300 and YY1 (Figure 4L), and immunofluorescence staining further confirmed their intracellular co‐localization (Figure 4M).

To determine whether p300 regulates YY1 lactylation in a K183‐dependent manner, BMSCs expressing Flag‐YY1‐WT or Flag‐YY1‐K183R were subjected to p300 overexpression or knockdown. Following anti‐Flag immunoprecipitation, YY1 lactylation was assessed by immunoblotting with a pan‐Kla antibody. Compared with the corresponding control, p300 overexpression significantly increased YY1‐WT lactylation, whereas p300 knockdown markedly reduced it. In contrast, neither p300 overexpression nor p300 knockdown significantly altered the residual lactylation of YY1‐K183R (Figure 4N,O). These results support a role for p300 in regulating YY1‐associated lactylation signals in a manner influenced by K183.

We subsequently examined whether p300‐dependent YY1 lactylation contributes to DUSP26 transcriptional regulation. In p300‐knockdown BMSCs, YY1 overexpression no longer significantly increased DUSP26 mRNA or protein expression, as determined by RT‐qPCR and Western blotting, respectively (Figure 4P). Combined p300 and YY1 perturbation experiments showed that p300 knockdown attenuated YY1‐WT‐mediated DUSP26 upregulation, whereas p300 overexpression potentiated this effect. By contrast, neither p300 overexpression nor p300 knockdown significantly altered DUSP26 expression in cells expressing YY1‐K183R (Figure 4Q,R). These molecular changes were accompanied by corresponding functional effects. p300 knockdown reduced the increases in ALP staining intensity and mineralized nodule formation induced by YY1‐WT, whereas p300 overexpression further enhanced the osteogenic effects of YY1‐WT. Neither manipulation significantly altered ALP or ARS staining in cells expressing YY1‐K183R (Figure S2E,F). Enzyme‐linked immunosorbent assay (ELISA) measurements of pro‐angiogenic factors, including VEGF, ANG2, and SLIT3, showed a similar pattern (Figure S2G,H). Collectively, these findings support a p300‐dependent YY1 K183 lactylation‐DUSP26 transcriptional axis that promotes osteogenic differentiation and enhances the pro‐angiogenic paracrine activity of BMSCs.

### 2.5 Mitophagy Dysfunction Contributes to Impaired Osteogenesis Caused by DUSP26 Deficiency in BMSCs

Because DUSP26 has been reported to localize predominantly to the nucleus and mitochondria and to regulate mitochondrial function, we next investigated whether DUSP26 influences mitochondrial homeostasis in BMSCs (Figure 5A). Subcellular fractionation followed by Western blotting showed that DUSP26 was mainly localized in the nucleus and mitochondria (Figure 5B). Consistently, IF analysis demonstrated co‐localization of DUSP26 with mitochondrial signals (Figure 5C).

**FIGURE 5:**
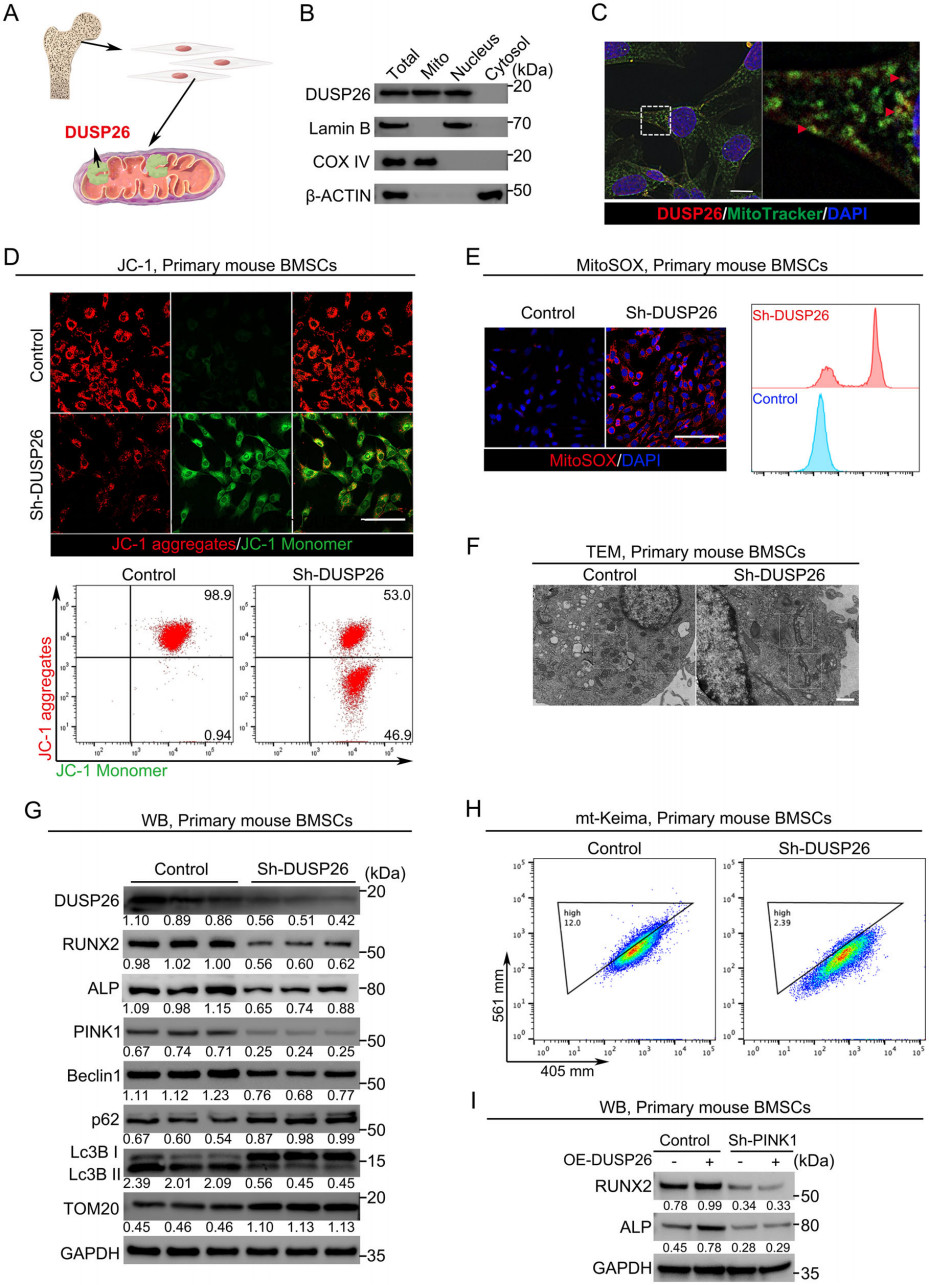
DUSP26 knockdown inhibits osteogenic differentiation of BMSCs by suppressing mitophagy. (A) Schematic diagram of mitochondrial localization of DUSP26 in BMSCs. (B, C) Western blotting (*n* = 3) and IF analysis were employed to evaluate the localization of DUSP26.The red arrow indicates the co‐localization of DUSP26 and MitoTracker. (C) Scale bar = 20 µm. (D–F) Staining and flow cytometry analysis of JC‐1 and MitoSOX, as well as Transmission electron microscopy(TEM), were used to assess mitochondrial damage in DUSP26‐knockdown BMSCs (*n* = 3). (D, E) Scale bar = 50 µm. (F) Scale bar = 1 µm. (G) Western blotting was used to analyze the effects of DUSP26 knockdown on mitophagy (*n* = 3). (H) Mt‐Keima‐based flow cytometric analysis was used to assess the effect of DUSP26 on mitophagy. (I) Western blotting analysis of the effects of DUSP26 overexpression on the osteogenic differentiation of PINK1‐knockdown BMSCs (*n* = 3). ^**^ *p* < 0.01, ^***^ *p* < 0.001,^****^ *p* < 0.0001, ns, not significant.

To assess mitochondrial function, JC‐1 staining was performed. Confocal microscopy showed that DUSP26 knockdown decreased the red fluorescence of JC‐1 aggregates and increased the green fluorescence of JC‐1 monomers, indicating a reduction in mitochondrial membrane potential (ΔΨm). Flow cytometry analysis further confirmed the decrease in ΔΨm caused by DUSP26 deficiency (Figure 5D). MitoSOX staining revealed that DUSP26 knockdown markedly increased mitochondrial ROS levels, which was further validated by flow cytometry (Figure 5E). TEM showed that, compared with the vector group, mitochondria in shDUSP26‐treated BMSCs were enlarged and exhibited loss of the double membrane, indistinct cristae, and blurred boundaries (Figure 5F). These findings indicate that DUSP26 is required for maintaining normal mitochondrial structure and function in BMSCs.

We next examined the expression of mitophagy‐related proteins. Western blotting showed that DUSP26 knockdown decreased the expression of PINK1 and Beclin1, reduced the microtubule‐associated protein 1 light chain 3 beta(LC3B)‐II/LC3B‐I ratio, and increased the levels of p62 and translocase of the outer mitochondrial membrane 20 (TOM20) in BMSCs (Figure 5G). In addition, the monomeric red fluorescent protein‐green fluorescent protein‐microtubule‐associated protein 1 light chain 3 (mRFP‐GFP‐LC3) assay demonstrated that DUSP26 knockdown significantly reduced autophagic flux, as reflected by decreased accumulation of fluorescent puncta (Figure S3A). To further assess the role of DUSP26 in regulating mitophagy in BMSCs, mt‐Keima–based flow cytometry was used to quantify mitochondrial delivery to acidic lysosomal compartments. mt‐Keima is targeted to the mitochondrial matrix and exhibits pH‐dependent excitation properties, enabling the assessment of mitochondrial delivery to acidic lysosomes. Compared with the control group, the shDUSP26 group exhibited a significantly lower proportion of cells positive for acidic mt‐Keima fluorescence, accompanied by a marked reduction in the acidic‐to‐neutral mt‐Keima fluorescence ratio (Figure 5H). Together, these findings suggest that DUSP26 knockdown reduces mitochondrial delivery to acidic lysosomal compartments during osteogenic differentiation.

To further determine whether mitophagy mediates the effects of DUSP26 on BMSC osteogenic differentiation and pro‐angiogenic factor secretion, BMSCs were first transduced with an shRNA targeting PINK1, a key regulator of mitophagy, and subsequently transfected with a DUSP26‐overexpression plasmid. Following osteogenic induction, the protein levels of the osteogenic markers RUNX2 and ALP were assessed by Western blotting. In BMSCs without PINK1 knockdown, DUSP26 overexpression significantly increased RUNX2 and ALP protein levels. However, these increases were no longer evident in PINK1‐knockdown BMSCs (Figure 5I). Consistently, ELISA analysis of culture supernatants showed that the DUSP26‐induced increase in pro‐angiogenic factor secretion was also abolished following PINK1 knockdown (Figure S3B). In vivo, IF analysis showed reduced PINK1 staining in leptin receptor (LepR)‐positive cells in bone tissue from shDUSP26‐treated mice (Figure S3C). Collectively, these results demonstrate that PINK1 knockdown markedly attenuates the ability of DUSP26 to promote osteogenic differentiation and pro‐angiogenic factor secretion in BMSCs, indicating that these effects are mediated by mitophagy.

### 2.6 DUSP26 Binds to the Core Region of PMPCB Through its C‐Terminal Domain

To elucidate the molecular mechanism by which DUSP26 regulates mitophagy, we intersected proteins predicted to interact with DUSP26 in the BIOGRID database with mitochondria‐related proteins annotated in MitoCarta. This analysis identified two candidate proteins, mitochondrial processing peptidase subunit alpha (PMPCA) and PMPCB, that were both associated with mitochondrial function and predicted to interact with DUSP26 (Figure 6A). Co‐IP assays showed that no detectable association between DUSP26 and PMPCA was observed, but interacted specifically with PMPCB (Figure 6B). IF staining further confirmed the co‐localization of DUSP26 and PMPCB in BMSCs (Figure 6C).

**FIGURE 6:**
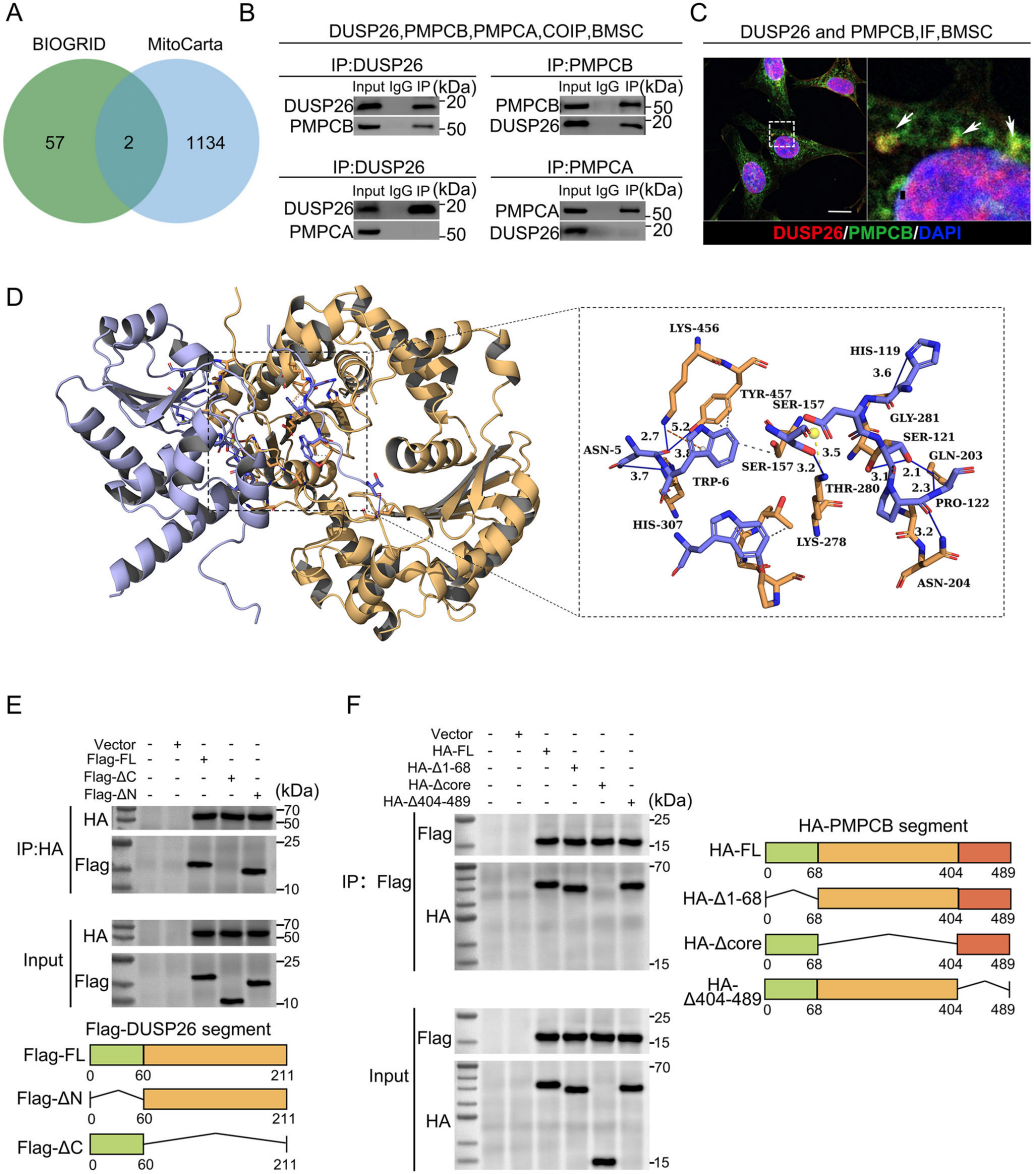
DUSP26 binds to PMPCB. (A) Identification of candidate DUSP26‐interacting mitochondrial proteins. (B–D) Co‐IP, IF analysis, and three‐dimensional molecular docking were used to evaluate the interaction between PMPCB and DUSP26. (C) The white arrow indicates the co‐localization site. (C) Scale bar = 10 µm. (E, F) Co‐IP analysis of the interactions involving DUSP26 truncation mutants (E) and PMPCB deletion mutants (F). ^**^ *p* < 0.01, ^***^ *p* < 0.001,^****^ *p* < 0.0001, ns, not significant.

Molecular docking analysis supported a structural basis for the interaction between DUSP26 and PMPCB (Figure 6D), and molecular mechanics/generalized born surface area calculations yielded a binding free energy of −87.36 kcal/mol, indicating the formation of a stable complex. To further define the regions responsible for this interaction, DUSP26 truncation mutants lacking either the N‐terminal region (Δ1–60) or the C‐terminal region (Δ60–211) were generated. Co‐IP assays showed that the C‐terminal region of DUSP26 mediated its interaction with PMPCB (Figure 6E). Correspondingly, PMPCB deletion mutants, including Δ1–68, Δcore, and Δ404–489, were constructed, and Co‐IP analysis showed that the core region of PMPCB was required for binding to DUSP26 (Figure 6F). These results indicate that DUSP26 binds to PMPCB through its C‐terminal domain, whereas the core region of PMPCB mediates this interaction.

### 2.7 PMPCB Regulates Osteogenic Differentiation and Proangiogenic Factor Secretion in BMSCs by Inhibiting PINK1‐Dependent Mitophagy

To determine whether PMPCB participates in the osteogenesis of BMSCs, we first examined its expression during osteogenic differentiation. RT‐qPCR and Western blotting showed that PMPCB mRNA and protein levels did not change significantly during osteogenic differentiation of BMSCs (Figure 7A,B). We then overexpressed PMPCB in BMSCs using plasmid transfection. Western blotting showed that PMPCB overexpression decreased the protein levels of ALP and RUNX2 (Figure 7C). Consistently, ALP and ARS staining intensities were reduced following PMPCB overexpression (Figure 7D). ELISA further showed that the concentrations of VEGF, ANG2, and SLIT3 in the culture medium were decreased after PMPCB overexpression (Figure 7E). In contrast, PMPCB knockdown by shRNA increased the protein expression of ALP and RUNX2 (Figure 7F), enhanced ALP and ARS staining (Figure 7G), and increased the concentrations of VEGF, ANG2, and SLIT3 in the culture medium (Figure 7H). These findings indicate that PMPCB negatively regulates osteogenic differentiation and the pro‐angiogenic secretory profile of BMSCs.

**FIGURE 7:**
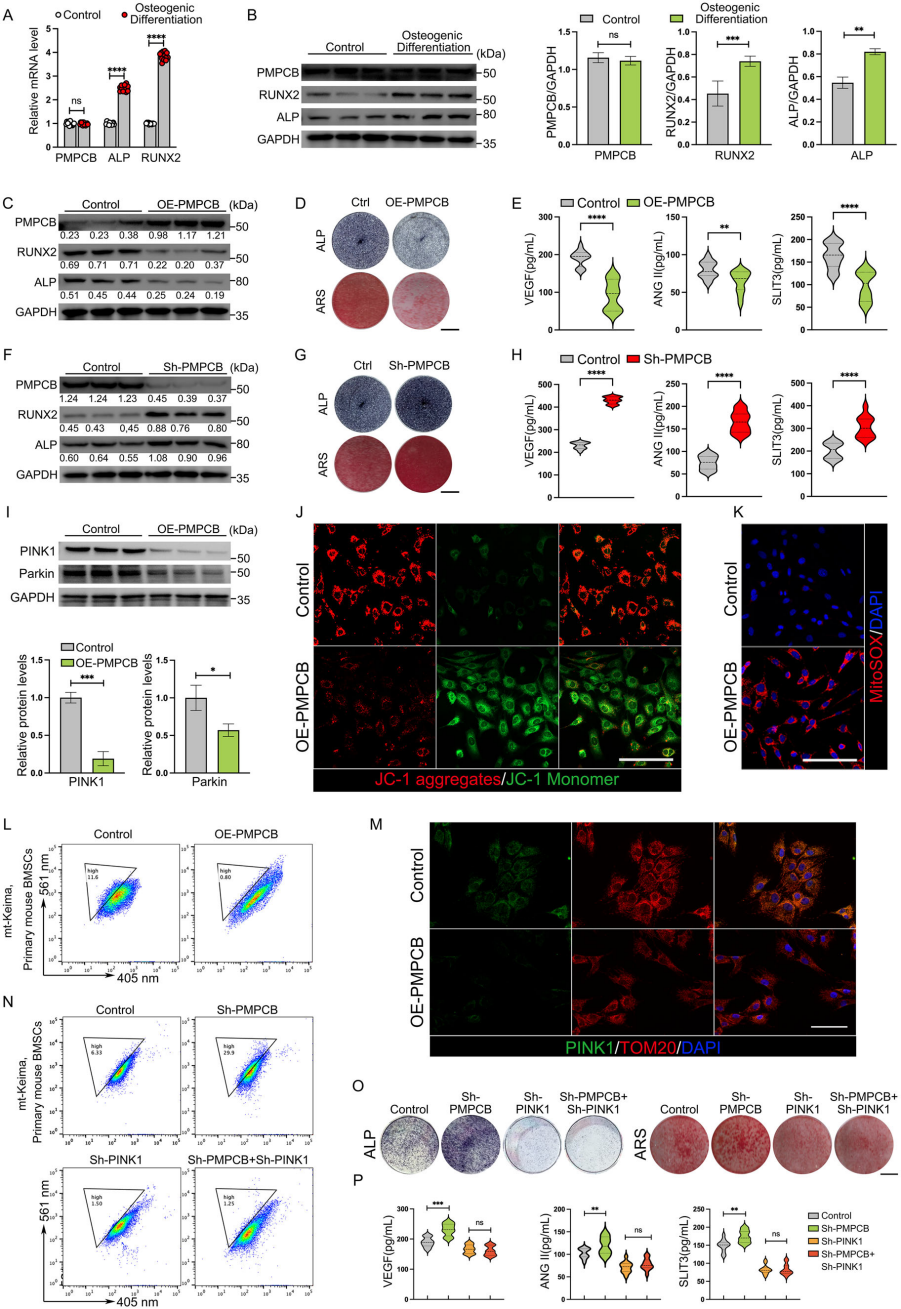
Effects of PMPCB on BMSCs. (A, B) RT‐qPCR (*n* = 12) and Western blotting (*n* = 3) analyses of PMPCB expression during osteogenic differentiation of BMSCs. (C–E) Western blotting (*n* = 3), ALP staining, ARS staining (*n* = 3) and ELISA (*n* = 12)were used to evaluate osteogenic differentiation and proangiogenic factor secretion in PMPCB‐overexpressing BMSCs. (D) Scale bar = 5 mm. (F–H) Western blotting (*n* = 3), ALP staining, ARS staining (*n* = 3), and ELISA (*n* = 12)were used to evaluate osteogenic differentiation and proangiogenic factor secretion in PMPCB‐knockdown BMSCs. (G) Scale bar = 5 mm. (I) Western blotting was used to assess PINK1 and Parkin expression in PMPCB‐overexpressing BMSCs. (*n* = 3) (J, K) JC‐1 and MitoSOX staining were employed to evaluate the effects of PMPCB overexpression on mitochondria. Scale bar = 50 µm. (L) Flow cytometry was used to assess mitophagy levels in PMPCB‐overexpressing BMSCs. (*n* = 3) (M) Immunofluorescence staining was used to assess the colocalization of PINK1 with TOM20. Scale bar = 50 µm. (N–P) Flow cytometry, ALP and ARS staining, and ELISA were used to assess the effects of PMPCB knockdown on mitophagy, osteogenic differentiation, and proangiogenic factor secretion in PINK1‐knockdown BMSCs. ^**^ *p* < 0.01, ^***^ *p* < 0.001,^****^ *p* < 0.0001, ns, not significant.

Previous studies have shown that PMPCB forms the mitochondrial processing peptidase (MPP) complex with PMPCA and participates in the regulation of PINK1/Parkin signaling [22]. To determine whether PMPCB regulates mitophagy in BMSCs through this pathway, we overexpressed PMPCB in BMSCs and assessed full‐length PINK1 and Parkin protein levels by Western blotting. Compared with the control group, PMPCB overexpression significantly reduced the levels of both full‐length PINK1 and Parkin (Figure 7I), suggesting that PMPCB overexpression suppresses PINK1/Parkin signaling.

We next examined the effects of PMPCB overexpression on mitochondrial function in BMSCs. JC‐1 staining revealed a significant reduction in the red‐to‐green fluorescence ratio following PMPCB overexpression, indicating a decrease in mitochondrial membrane potential (Figure 7J). MitoSOX staining further showed that PMPCB overexpression significantly increased mitochondrial ROS levels (Figure 7K). These findings indicate that PMPCB overexpression impairs mitochondrial function in BMSCs.

To further assess mitophagy, we transfected control and PMPCB‐overexpressing BMSCs with an mt‐Keima reporter construct and analyzed the cells by flow cytometry. Compared with the control group, PMPCB‐overexpressing cells exhibited a significantly reduced acidic mt‐Keima signal, suggesting decreased mitochondrial delivery to acidic lysosomes and impaired mitophagy (Figure 7L). In addition, dual immunofluorescence staining for PINK1 and the mitochondrial outer membrane marker TOM20 revealed significantly reduced colocalization following PMPCB overexpression, indicating a decrease in mitochondria‐associated PINK1 signal (Figure 7M). Collectively, these findings suggest that PMPCB overexpression suppresses PINK1/Parkin signaling and mitophagy and is accompanied by mitochondrial dysfunction.

To investigate whether PMPCB‐mediated regulation of mitophagy depends on PINK1, we knocked down PMPCB or PINK1 individually, or both simultaneously, in BMSCs, with appropriate controls. Cells in each group were subsequently transfected with the mt‐Keima reporter construct and analyzed by flow cytometry. PMPCB knockdown alone significantly increased the acidic mt‐Keima signal, whereas PINK1 knockdown alone significantly reduced it. In PINK1‐knockdown cells, additional PMPCB knockdown failed to produce the enhancement observed with PMPCB knockdown alone (Figure 7N). These findings support a requirement for PINK1 in the mitophagy‐promoting effect of PMPCB knockdown.

We further assessed the effects of these interventions on osteogenic differentiation and pro‐angiogenic factor secretion in BMSCs. Following osteogenic induction, ALP and ARS staining showed that PMPCB knockdown significantly increased ALP staining intensity and mineralized nodule formation in BMSCs without PINK1 knockdown. In contrast, no apparent enhancement of these osteogenic phenotypes was observed following PMPCB knockdown in PINK1‐knockdown cells (Figure 7O). CM generated by equal numbers of cells over the same culture period were also collected, and VEGF, ANG2, and SLIT3 levels were measured by ELISA. PMPCB knockdown significantly increased the levels of these factors in BMSCs without PINK1 knockdown, whereas no corresponding increases were observed in PINK1‐knockdown cells (Figure 7P). Together with the mt‐Keima results, these findings support the involvement of PINK1‐dependent mitophagy in the regulation of BMSC osteogenic differentiation and pro‐angiogenic factor secretion by PMPCB.

### 2.8 DUSP26 Dephosphorylates PMPCB at Y141 to Regulate Osteogenesis and Angiogenesis

To elucidate the molecular mechanism by which DUSP26 regulates PMPCB, we first examined whether DUSP26 affects PMPCB protein stability or mitochondrial localization. Following treatment with cycloheximide (CHX) to inhibit de novo protein synthesis, cells were collected at different time points, and the remaining PMPCB protein levels were assessed by Western blotting. Neither DUSP26 knockdown nor overexpression significantly altered the decline in PMPCB abundance over the examined time points (Figure S4A). In addition, double immunofluorescence staining for PMPCB and a mitochondrial marker revealed no apparent changes in the mitochondrial localization of PMPCB following modulation of DUSP26 expression (Figure S4B). These results indicate that altering DUSP26 expression did not appreciably affect PMPCB protein stability or mitochondrial localization under the conditions examined.

To assess the contribution of DUSP26 catalytic activity to PMPCB regulation, we first compared the phosphatase activities of recombinant wild‐type DUSP26 (WT) and the catalytically inactive C152S mutant. Purified DUSP26 WT and C152S were compared at matched protein amounts using the SensoLyte pNPP Protein Phosphatase Assay, with a reaction mixture lacking recombinant protein serving as the blank control. Phosphatase activity was determined by measuring the p‐nitrophenol generated through pNPP hydrolysis. DUSP26 WT catalyzed pNPP hydrolysis in a time‐ and dose‐dependent manner, whereas the activity of C152S remained close to the blank control level (Figure 8A–C). These findings support Cys152 as a key residue required for DUSP26 catalytic activity.

**FIGURE 8:**
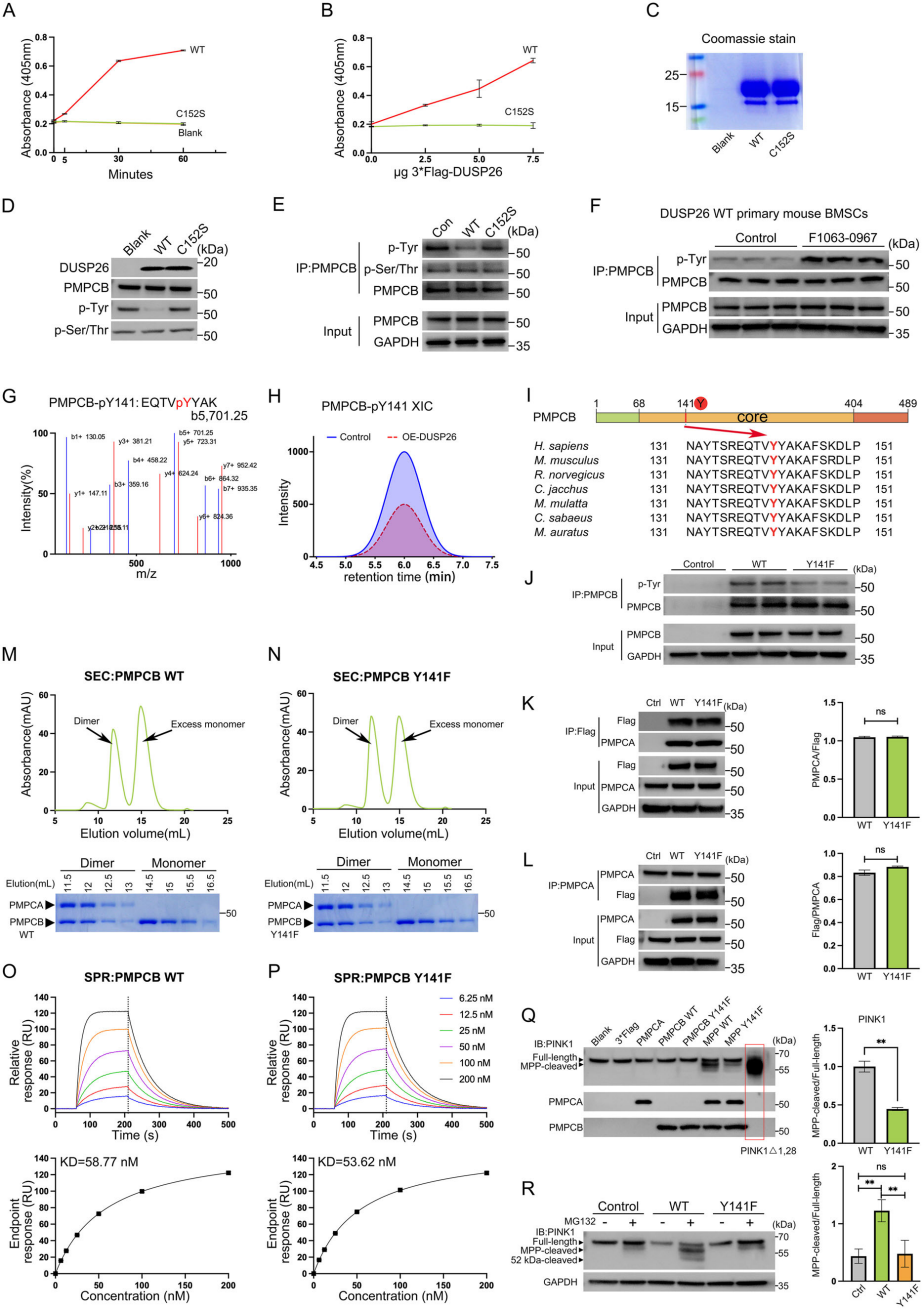
DUSP26 dephosphorylates the PMPCB Y141 site. (A) Bacterially expressed and purified FLAG‐DUSP26 or the FLAG‐DUSP26(C152S) mutant (15 µg) was incubated at 37°C for 0–60 min, and phosphatase activity toward pNPP was determined by quantifying the colorimetric signal at 405 nm. (*n* = 3). (B) The reaction conditions were the same as those in panel A, except that 0, 2.5, 5, or 7.5 µg of FLAG‐DUSP26 or FLAG‐DUSP26(C152S) was added to each reaction mixture, followed by incubation at 37°C for 30 min. (*n* = 3). (C) Coomassie Brilliant Blue staining of purified FLAG‐DUSP26 and FLAG‐DUSP26(C152S) proteins. (D) The in vitro dephosphorylation assay involved incubating purified PMPCB (1 µg) with purified FLAG‐DUSP26 or FLAG‐DUSP26(C152S) (0.25 µg) for 30 min. Western blotting subsequently assessed PMPCB phosphotyrosine and phosphoserine/threonine levels. (E) Western blotting assessed the effects of FLAG‐DUSP26 or FLAG‐DUSP26(C152S) expression on PMPCB phosphorylation in BMSCs. (F) Western blotting assessed the effect of F1063‐0967 on PMPCB phosphorylation in BMSCs transfected with DUSP26 WT. (G, H) Mass spectrometry identified PMPCB phosphorylation sites and assessed changes in the relative abundance of PMPCB phosphopeptides in BMSCs transfected with FLAG‐DUSP26 or FLAG‐DUSP26(C152S). (I) Cross‐species conservation of the PMPCB Y141 residue. (J) Immunoprecipitation followed by Western blotting assessed the tyrosine phosphorylation levels of PMPCB‐WT and PMPCB‐Y141F. (K, L) Forward and reciprocal Co‐IP analyses assessed the interaction of PMPCA with PMPCB‐WT or PMPCB‐Y141F in BMSCs. (*n* = 3). (M, N) Size‐exclusion chromatography generated elution profiles for WT‐MPP and Y141F‐MPP. Sodium dodecyl sulfate‐polyacrylamide gel electrophoresis (SDS‐PAGE) resolved proteins in fractions from both peaks, and Coomassie Brilliant Blue staining visualized the bands. (O, P) Surface plasmon resonance (SPR) analysis compared the binding of purified PMPCB‐WT and PMPCB‐Y141F to recombinant mouse PMPCA immobilized on the sensor surface. Both proteins generated concentration‐dependent binding responses at 6.25, 12.5, 25, 50, 100, and 200 nm. Kinetic analysis yielded the association rate constant (k_a), dissociation rate constant (k_d), and equilibrium dissociation constant (K_D) for each interaction. (Q) A cell‐free MPP cleavage assay compared the ability of purified WT‐MPP and Y141F‐MPP to process recombinant full‐length mouse PINK1. (*n* = 3). (R) Western blotting assessed the effects of PMPCB‐WT and PMPCB‐Y141F expression on PINK1 processing in BMSCs. (*n* = 3)^**^ *p* < 0.01, ^***^ *p* < 0.001,^****^ *p* < 0.0001, ns, not significant.

We next used PMPCB immunopurified from BMSCs as the substrate in a cell‐free phosphatase assay. Equal amounts of purified DUSP26 WT or C152S were added to the reaction, and a PMPCB‐containing reaction without DUSP26 served as the control. DUSP26 WT significantly reduced the overall tyrosine phosphorylation signal of PMPCB without appreciably affecting its serine/threonine phosphorylation. In contrast, C152S did not appreciably alter either phosphorylation signal (Figure 8D). These results support the ability of DUSP26 to reduce PMPCB tyrosine phosphorylation in a catalytic activity‐dependent manner in this cell‐free system.

To determine whether this regulation also occurs in cells, DUSP26 WT or C152S was overexpressed in BMSCs, and endogenous PMPCB was enriched by immunoprecipitation. The immunoprecipitates were analyzed by Western blotting using pan‐phosphotyrosine and pan‐phosphoserine/threonine antibodies. DUSP26 WT significantly reduced PMPCB tyrosine phosphorylation without appreciably affecting its serine/threonine phosphorylation, whereas C152S did not significantly alter either phosphorylation signal (Figure 8E). Pharmacological treatment of DUSP26 WT‐overexpressing BMSCs with F1063‐0967 significantly counteracted the reduction in PMPCB tyrosine phosphorylation induced by DUSP26 WT (Figure 8F). Together with the cell‐free assay results, these findings support the involvement of DUSP26 phosphatase activity in the regulation of PMPCB tyrosine phosphorylation.

To identify PMPCB phosphorylation sites associated with DUSP26 regulation, Endogenous PMPCB was enriched by immunoprecipitation from BMSCs transfected with DUSP26 WT or C152S for subsequent liquid chromatography‐tandem mass spectrometry (LC‐MS/MS) analysis. A phosphorylated PMPCB peptide with the sequence EQTVYYAK was detected, and site localization assigned the phosphorylation to Y141, yielding EQTVpYYAK (Figure 8G). After treatment, the relative abundance of this phosphorylated peptide in the WT transfection group was reduced by approximately 50% compared to the C152S group (Figure 8H). Multispecies sequence alignment further showed that the residue corresponding to Y141 was conserved as tyrosine in all seven mammalian species examined (Figure 8I). These findings support Y141 as an endogenous phosphorylation site in PMPCB in BMSCs and suggest that its phosphorylation is regulated by DUSP26. To assess the contribution of Y141 to the overall tyrosine phosphorylation signal of PMPCB, we generated PMPCB‐WT and the non‐phosphorylatable PMPCB‐Y141F mutant, both carrying the same C‐terminal 3×FLAG tag. Western blotting showed that the overall tyrosine phosphorylation signal was lower for PMPCB‐Y141F than for PMPCB‐WT (Figure 8J). Together with the LC–MS/MS results, these data support a contribution of Y141 to the detected tyrosine phosphorylation signal of PMPCB.

We next investigated the functional significance of Y141 by examining whether the Y141F substitution affects the interaction between PMPCB and PMPCA. In the forward Co‐IP assay, an anti‐FLAG antibody was used to precipitate PMPCB‐WT or PMPCB‐Y141F, and co‐precipitated endogenous PMPCA was detected. PMPCA was detected in both immunoprecipitates, with no significant difference in its relative association with PMPCB‐WT and PMPCB‐Y141F (Figure 8K). In the reciprocal assay, PMPCA was immunoprecipitated, and co‐precipitated FLAG‐tagged PMPCB was detected. After normalization of the FLAG signal to immunoprecipitated PMPCA, no significant difference in relative PMPCB recovery was observed between the two groups (Figure 8L). These results suggest that the Y141F substitution did not produce a detectable change in the PMPCA–PMPCB interaction under the conditions examined.

To further assess MPP complex assembly, mitochondria were isolated from BMSCs expressing FLAG‐tagged PMPCB‐WT or PMPCB‐Y141F, followed by FLAG affinity purification and size‐exclusion chromatography (SEC). Both preparations yielded a major elution peak with an apparent molecular mass of approximately 105–110 kDa and a secondary peak at approximately 50–55 kDa. SDS‐PAGE analysis of the corresponding fractions supported the assignment of these peaks to the MPP heterodimer and excess PMPCB WT or Y141F monomer, respectively (Figure 8M,N).

SPR was subsequently used to compare the binding of PMPCA to PMPCB‐WT and PMPCB‐Y141F. Recombinant mouse PMPCA was immobilized on the sensor surface, and PMPCB‐WT or PMPCB‐Y141F was passed over the surface at different concentrations. Both proteins produced concentration‐dependent binding responses. For PMPCB‐WT, the equilibrium dissociation constant (K_D) was 58.77 nm, the association rate constant (k_a) was 2.900 × 10^5^ M^−1^ s^−1^, and the dissociation rate constant (k_d) was 1.702 × 10^−2^ s^−1^. The corresponding values for PMPCB‐Y141F were 53.62 nm, 2.878 × 10^5^ M^−1^ s^−1^, and 1.502 × 10^−2^ s^−1^, respectively (Figure 8O,P). These binding parameters were similar between the two proteins. Together with the reciprocal Co‐IP and SEC results, these findings suggest that the Y141F substitution did not markedly affect PMPCA binding or MPP complex assembly.

Having observed no apparent differences in subunit association or complex assembly, we next compared the ability of MPP complexes containing PMPCB‐WT or PMPCB‐Y141F to process PINK1. SEC‐purified PMPCB‐WT/PMPCA heterodimers (WT‐MPP) or PMPCB‐Y141F/PMPCA heterodimers (Y141F‐MPP) were incubated with full‐length recombinant mouse PINK1, with PMPCA or PMPCB alone included as single‐subunit controls. After 60 min of incubation, WT‐MPP reduced full‐length PINK1 abundance, accompanied by the appearance of an approximately 60‐kDa processed product. In contrast, PINK1 processing was markedly reduced in the Y141F‐MPP reaction (Figure 8Q). These results indicate that the Y141F substitution reduces the ability of MPP to process PINK1 in vitro. We subsequently overexpressed PMPCB‐WT or PMPCB‐Y141F in BMSCs to assess their effects on cellular PINK1 processing. Western blotting showed that, compared with control cells, PMPCB‐WT overexpression reduced the relative abundance of full‐length PINK1 and increased the signal ratio of the putative MPP‐processed PINK1 band to full‐length PINK1. In contrast, PMPCB‐Y141F‐expressing cells retained more full‐length PINK1 and exhibited a significantly lower processed/full‐length PINK1 ratio than PMPCB‐WT‐expressing cells (Figure 8R). These cellular changes were consistent with the in vitro processing results, supporting a functional contribution of Y141 to PMPCB‐dependent PINK1 processing.

To link the catalytic activity of DUSP26 to its regulation of PMPCB‐associated cellular functions, we introduced DUSP26 WT or C152S into PMPCB‐overexpressing BMSCs. Compared with PMPCB overexpression alone, co‐expression of DUSP26 WT significantly reversed the suppression of mitophagy detected by mt‐Keima analysis, whereas DUSP26 C152S did not (Figure S4C). Consistent results were obtained using ALP staining, ARS staining, and ELISA. DUSP26 WT significantly counteracted the PMPCB‐induced reductions in osteogenic differentiation and proangiogenic factor secretion, whereas C152S failed to reverse these effects (Figure S4D,E). To further investigate the contribution of PMPCB Y141 to DUSP26‐dependent regulation, BMSCs expressing PMPCB‐WT or PMPCB‐Y141F were subsequently transfected with shDUSP26. In PMPCB‐WT‐expressing cells, DUSP26 knockdown significantly increased the processed/full‐length PINK1 ratio compared with the corresponding control, suggesting enhanced PINK1 processing (Figure S4F). In contrast, DUSP26 knockdown did not appreciably alter this ratio in PMPCB‐Y141F‐expressing cells. In PMPCB‐WT‐expressing BMSCs, DUSP26 knockdown reduced the acidic mt‐Keima‐positive population, ALP/ARS staining, and the concentrations of proangiogenic factors. In PMPCB‐Y141F‐expressing BMSCs, no appreciable additional effect of DUSP26 knockdown was observed (Figure S4G–I).

Together, these findings support a model in which DUSP26 reduces PMPCB tyrosine phosphorylation through its phosphatase activity, thereby limiting MPP‐mediated PINK1 processing and promoting mitophagy, osteogenic differentiation, and proangiogenic factor secretion in BMSCs. The Y141F experiments further implicate Y141 in this regulatory mechanism.

### 2.9 Engineered Exosomes Carrying a RUNX2 Promoter‐Driven DUSP26 Plasmid Alleviate Osteoporosis by Promoting Osteogenesis and Type H Vessel Formation

We next explored the therapeutic potential of DUSP26 in osteoporosis. To this end, we designed a DUSP26 plasmid driven by the osteogenic‐specific RUNX2 promoter and used BMSC‐derived exosomes as the delivery vehicle to construct a targeted therapeutic system, termed RUNX2‐DUSP26‐EXO. Previous studies have shown that the promoter region from −965 to −16 of RUNX2 exhibits high activity during early osteogenic differentiation of BMSCs [23]; therefore, this sequence was selected to drive DUSP26 expression. The plasmid was encapsulated into exosomes by electroporation. TEM showed that the engineered exosomes retained the typical bilayer membrane structure, with no marked morphological difference compared with unloaded exosomes, and the diameter of the engineered exosomes was approximately 100 nm and did not differ significantly from that of conventional exosomes (Figure 9A). Nano‐flow cytometry further showed a slight increase in particle size and a decrease in zeta potential after plasmid loading (Figure 9B). To further characterize the isolated extracellular vesicles, western blotting was performed to assess exosome‐associated marker proteins. cluster of differentiation 9 (CD9), cluster of differentiation 63 (CD63), and tumor susceptibility gene 101 (TSG101) were detected in both the EXO and RUNX2‐DUSP26‐EXO preparations, whereas the endoplasmic reticulum marker calnexin was undetectable. This protein profile indicated that the isolated vesicles were enriched in canonical exosome‐associated proteins and contained minimal contamination from intracellular membrane components (Figure 9C). Together with the TEM observations and particle‐size analysis, these findings support the successful isolation of BMSC‐derived exosomes and the production of engineered exosomes.

**FIGURE 9:**
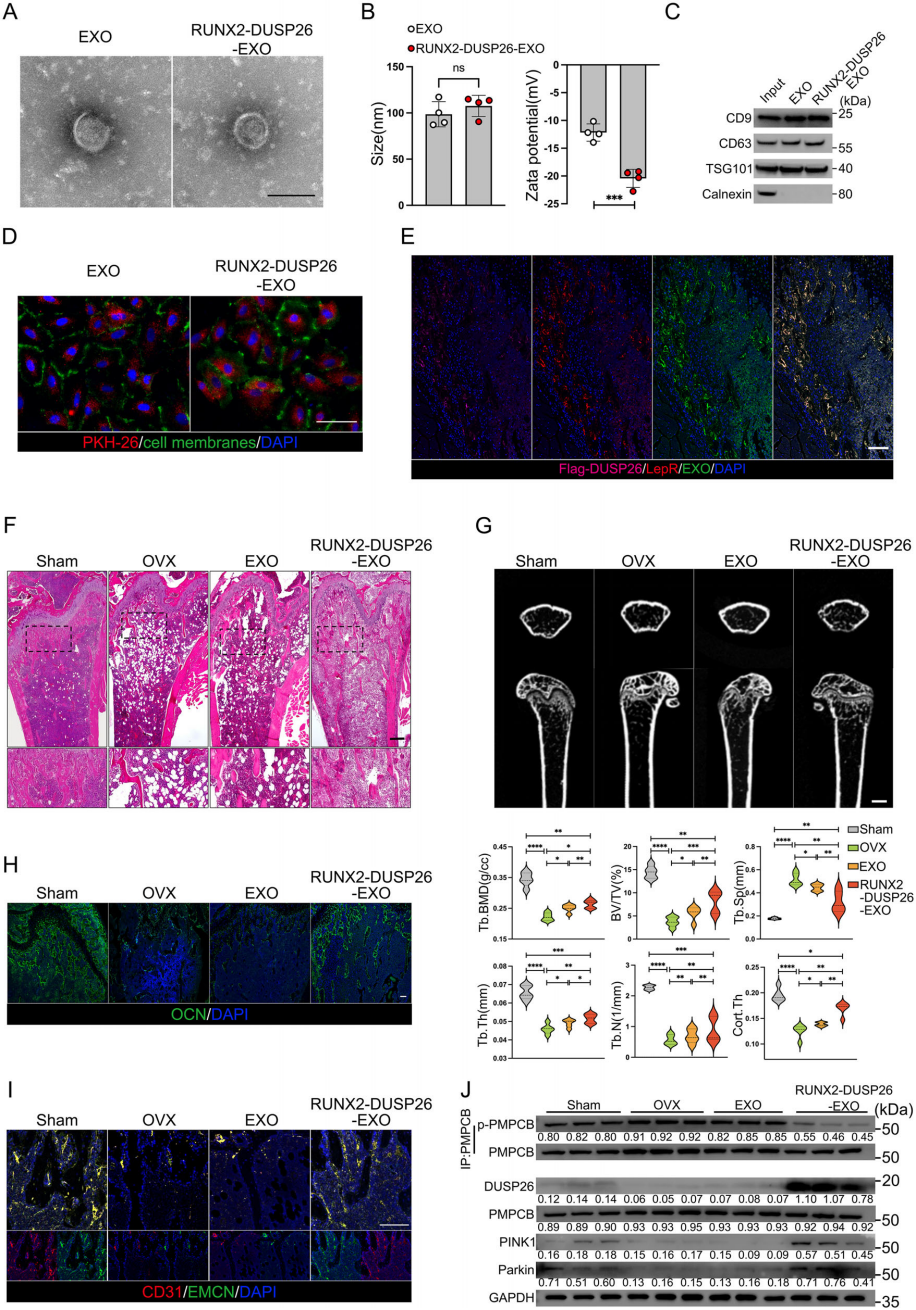
Characterization and in vivo evaluation of RUNX2‐DUSP26‐EXO. (A) Analysis of exosome morphology employing TEM. Scale bar = 100nm. (B)Measurement of size and zeta potential of EXO and RUNX2‐DUSP26‐EXO using nanofluorescence flow cytometry. (C) Western blotting assessed the presence of EXO‐associated marker proteins. (D) Confocal microscopy was used to observe the uptake of PKH‐26‐labeled exosomes by BMSCs. Scale bar = 50 µm. (E) Confocal imaging of PKH‐67‐labeled exosome distribution and FLAG‐DUSP26 expression in bone tissue. Scale bar = 100 µm. (F, G) H&E staining and Micro‐CT were employed to evaluate the therapeutic efficacy of RUNX2‐DUSP26‐EXO. (F) Scale bar = 200 µm. (G) Scale bar = 500 µm. (H, I) IF staining was used to assess OCN expression (H) and CD31/EMCN‐positive vessels (I) in bone tissue. Scale bar = 200 µm. (J) Western blotting was used to evaluate the phosphorylation levels of PMPCB and mitophagy levels in bone tissue after treatment with RUNX2‐DUSP26‐EXO.

To evaluate encapsulation efficiency, the Quant‐iT PicoGreen double‐stranded deoxyribonucleic acid(dsDNA) assay was performed. A standard curve was generated using serial dilutions of plasmid DNA, and fluorescence intensity was measured at excitation/emission wavelengths of 480/520 nm. The standard curve showed a linear fit with a coefficient of determination (R^2^) of 0.9898. The encapsulation efficiency, calculated as the proportion of plasmid DNA successfully loaded relative to the total input DNA, was 9.4% ± 0.8%, supporting the feasibility of this delivery strategy (Figure S5A). To assess cellular uptake, exosomes were labeled with PKH‐26 and BMSC membranes were labeled with PKH‐67. Confocal microscopy showed that BMSCs efficiently internalized the engineered exosomes (Figure 9D).

To assess the cell‐type‐dependent expression of the delivered DUSP26 transgene, BMSCs, HUVECs, and bone marrow‐derived macrophages (BMDMs) were treated with RUNX2‐DUSP26‐EXO. DUSP26 mRNA expression was more strongly increased in BMSCs than in HUVECs or BMDMs, consistent with preferential transgene expression in BMSCs under these experimental conditions (Figure S5B). In addition, RUNX2‐DUSP26‐EXO significantly increased DUSP26 mRNA expression in BMSCs compared with EXO, although the increase was slightly lower than that induced by CMV‐DUSP26‐EXO (Figure S5C). In vivo biodistribution and transgene‐expression analyses showed that PKH‐67‐labeled exosomes were taken up by multiple intramedullary cell types, whereas Flag‐DUSP26 expression was enriched in LepR‐positive osteogenic progenitor cells. These findings suggest preferential osteogenic‐lineage transgene expression (Figure 9E). Flow cytometric analysis of single‐cell suspensions prepared from bone marrow and major organs detected EXO signals in multiple bone marrow cell populations and in cells from some major organs. These findings indicate that uptake of BMSC‐derived extracellular vesicles was not strictly specific to BMSCs. Despite this broad distribution, FLAG‐DUSP26 signals were undetectable or very low in non‐osteogenic bone marrow cells and cells from major organs. In contrast, FLAG‐DUSP26 was detected predominantly in LepR^+^ osteoprogenitors (Figure S5D,E). Thus, EXO uptake occurred across multiple cell types, whereas the RUNX2 promoter largely restricted DUSP26 expression to osteogenic‐lineage cells.

We then evaluated the biosafety of RUNX2‐DUSP26‐EXO. H&E staining showed no obvious inflammation or structural damage in major organs, including the heart, liver, spleen, lung, kidney, and brain (Figure S5F). Blood biochemical analyses further indicated that RUNX2‐DUSP26‐EXO did not cause detectable systemic toxicity (Figure S6A).

To assess therapeutic efficacy in vivo, an ovariectomy (OVX)‐induced osteoporosis model was established in 8‐week‐old female mice. OVX mice were then treated with EXO or RUNX2‐DUSP26‐EXO (100 µg) via tail vein injection every 3 days for 6 weeks, whereas sham‐operated mice served as controls. No significant difference in body weight was observed among the groups during the treatment period (Figure S6B). H&E staining and micro‐CT analysis showed that RUNX2‐DUSP26‐EXO treatment increased Tb.N and Tb.Th, reduced Tb.Sp, and improved Tb.BMD compared with EXO treatment in OVX mice. However, this treatment only partially rescued OVX‐induced bone loss relative to the sham group (Figure 9F,G). Immunofluorescence staining further showed that RUNX2‐DUSP26‐EXO significantly increased OCN fluorescence intensity in OVX mice compared with the EXO group (Figure 9H and Figure S6C). Moreover, OVX mice treated with RUNX2‐DUSP26‐EXO exhibited a higher abundance of type H vessels than untreated OVX mice or EXO‐treated OVX mice, although this effect only partially restored the OVX‐induced reduction in type H vessels compared with sham controls (Figure 9I and Figure S6D). Finally, Western blotting showed that RUNX2‐DUSP26‐EXO increased DUSP26 expression, reduced PMPCB phosphorylation, and activated the PINK1/Parkin signaling pathway in bone tissue (Figure 9J). Together, these findings suggest that enhancing DUSP26 expression through engineered exosomes may represent a promising therapeutic strategy for osteoporosis by simultaneously promoting osteogenesis and type H angiogenesis.

## 3 Discussion

This study identifies DUSP26 as a regulator of osteogenic differentiation in BMSCs and supports its involvement in bone‐vascular coupling. DUSP26 expression was reduced in femoral tissues from osteoporotic mice and increased during osteogenic differentiation of BMSCs. DUSP26 knockdown suppressed osteogenic marker expression and mineralized nodule formation, whereas overexpression produced the opposite effects. Previous studies have reported that DUSP26 suppresses prostate cancer cell proliferation, migration, and invasion [24] and regulates kinesin family member 3‐associated transport and N‐cadherin‐mediated cell‐cell adhesion [25]. Our findings extend these observations to the regulation of BMSC function and bone homeostasis. Bone formation is closely linked to angiogenesis, and type H vessels contribute to the osteogenic vascular niche [26]. Signals including SLIT3 and VEGF participate in the regulation of type H vessels and bone formation [27]. In the present study, DUSP26 upregulation increased VEGF, ANG2, and SLIT3 levels in BMSC culture supernatants. CM from these cells enhanced HUVEC proliferation, migration, and network formation. These results support a role for DUSP26 in both BMSC osteogenic differentiation and pro‐angiogenic paracrine activity, although they do not establish the individual contribution of each secreted factor to the endothelial response.

Our in vivo data are consistent with a role for DUSP26 in maintaining bone formation and the associated vascular microenvironment. DUSP26 knockdown reduced trabecular bone mass, osteogenic marker expression, bone formation parameters, and type H vessel abundance. These parallel changes support an association between DUSP26‐dependent osteogenesis and vascular maintenance, but do not by themselves establish the direction of causality between the skeletal and vascular phenotypes. Previous studies have shown that osteoblast‐derived SLIT3 supports type H vessels and bone formation [28]. The phenotype observed after DUSP26 knockdown is therefore consistent with impaired osteoblast‐vascular communication, which warrants further cell‐type‐specific investigation.

Our findings support a role for p300‐associated YY1 lactylation in the transcriptional regulation of DUSP26 during osteogenic differentiation. Manipulation of p300 expression altered YY1‐associated lactylation signals and YY1‐dependent DUSP26 expression, while the K183R substitution attenuated these responses. ChIP‐qPCR and promoter‐reporter experiments support YY1 binding and transcriptional activity at the DUSP26 regulatory region. However, these assays do not directly establish that K183 lactylation increases YY1 occupancy at this locus. Lactylation is a post‐translational modification that can link metabolic state to gene regulation [29]. Endothelial cell‐derived lactate has been reported to promote histone lactylation and osteogenic gene expression in BMSCs, with reduced histone lactylation observed in BMSCs from patients with osteoporosis [30]. In microglia, p300‐associated YY1 K183 lactylation has also been linked to fibroblast growth factor 2 transcription and angiogenesis [21]. Our BMSC findings suggest that non‐histone lactylation‐associated regulation may contribute to osteogenic transcriptional programs. The lactate source and the modification‐specific effects of YY1 K183 nevertheless require further investigation in this system.

The mitochondrial findings provide a functional link between DUSP26 and BMSC osteogenesis. DUSP26 was detected in nuclear and mitochondrial fractions, although localization alone does not establish a nuclear transcriptional function. DUSP26 interacted with PMPCB, and the catalytic‐mutant, phosphatase, mass‐spectrometry, and mutational analyses support regulation of PMPCB tyrosine phosphorylation, with Y141 implicated as a relevant site. PMPCB has previously been linked to PINK1 processing [22]. In our experiments, reciprocal Co‐IP, size‐exclusion chromatography, and SPR detected no marked differences in PMPCA association or MPP complex assembly between PMPCB‐WT and PMPCB‐Y141F under the tested conditions. In contrast, MPP complexes containing PMPCB‐Y141F showed reduced PINK1 processing. Together with the cellular perturbation experiments, these results support a model in which DUSP26‐dependent regulation of PMPCB phosphorylation influences PINK1 processing without requiring a detectable change in MPP assembly. This model is consistent with the established role of PINK1/Parkin signaling in mitochondrial quality control [31]. PINK1 knockdown attenuated the osteogenic and pro‐angiogenic effects associated with DUSP26 or PMPCB manipulation, supporting the involvement of PINK1‐dependent mitophagy. These findings do not establish a change in PINK1 half‐life or the functional necessity of Parkin. DUSP26 knockdown also decreased mitochondrial membrane potential and increased ROS levels. Such mitochondrial impairment may contribute to the osteogenic phenotype, consistent with previous observations in DUSP26‐deficient neurons [19] and the broader involvement of mitochondrial dysfunction in osteoporosis [32].

We loaded BMSC‐derived exosomes with a RUNX2 promoter‐driven DUSP26 expression plasmid and found that treatment with these engineered exosomes attenuated bone loss in OVX mice. Treatment improved trabecular bone parameters and increased type H vessel abundance, supporting further investigation of this cell‐free approach. Vesicle uptake and transgene expression should be distinguished: uptake occurred across multiple cell types, whereas FLAG‐DUSP26 expression was preferentially detected in osteogenic‐lineage cells. Thus, the evidence supports preferential transgene expression rather than BMSC‐specific vesicle uptake. Mesenchymal stem cell‐derived extracellular vesicles are being investigated as delivery platforms for bone‐related diseases [33], but the present study did not directly compare their immunogenicity or safety with cell transplantation. The rationale for improving the skeletal vascular microenvironment is supported by recombinant SLIT3 studies reporting enhanced fracture healing and attenuation of OVX‐induced bone loss [28]. SLIT3‐loaded hydrogel microparticles have also been studied in tendon‐bone healing [34]. These precedents support further preclinical evaluation of DUSP26‐directed delivery, but do not establish clinical efficacy or superiority over existing osteoporosis treatments.

Several limitations remain. The conservation and clinical relevance of the proposed regulatory mechanism require validation in additional animal models and human samples. The YY1‐K183R substitution and p300 perturbations do not isolate lactylation from other modifications or functions of these proteins. Similarly, PMPCB‐Y141F supports a functional role for Y141 but cannot exclude effects of the substitution beyond loss of phosphorylation. The mechanisms linking mitochondrial regulation to individual secreted factors also remain unresolved, and HUVEC assays do not directly reproduce the properties of bone type H endothelial cells. For the delivery strategy, broader assessment of expression selectivity, dose‐response relationships, and long‐term efficacy is needed. Safety evaluation should extend beyond the measured organ histology and blood parameters. DUSP26 has been reported to promote aortic valve calcification [20], highlighting the need to distinguish skeletal benefits from potential extraskeletal effects. Preferential osteogenic‐lineage expression may be advantageous, but long‐term studies specifically assessing ectopic and valvular calcification remain necessary.

## 4 Conclusion

This study identifies DUSP26 as a regulator of BMSC osteogenic differentiation and pro‐angiogenic paracrine activity. The findings support a model linking p300‐associated YY1 lactylation and DUSP26 transcription to PMPCB phosphorylation, PINK1 processing, and mitophagy. Engineered BMSC‐derived exosomes carrying a RUNX2 promoter‐driven DUSP26 plasmid produced preferential transgene expression in osteogenic‐lineage cells and attenuated OVX‐induced bone loss. These results support further preclinical investigation of DUSP26‐directed approaches, with additional validation of modification‐specific mechanisms, expression selectivity, and long‐term safety.

## 5 Materials and Methods

### 5.1 Cell Culture and Treatment

In accordance with our prior studies [4], primary BMSCs were isolated from the tibia and femur of 4‐week‐old C57BL/6 J mice. Subsequently, they were cultured and passaged in Dulbecco's Modified Eagle's Medium/Ham's F–12 (DMEM/F12) (Gibco, USA). Moreover, 10% fetal bovine serum (FBS) (Cyagen, China) along with 100 U/mL penicillin and streptomycin were added, and the cells were incubated in an incubator (Thermo, USA) with constant temperature and humidity at 37°C, containing 95% air and 5% carbon dioxide. The adherent cells were regarded as BMSCs and underwent the first passage on day 5 (P1). Thereafter, these cells were subcultured once the confluence reached 80%. When the P3 BMSCs reached 80% confluence, they were utilized for subsequent experiments. For the BMSCs identification experiments conducted in a previous study, three–line induction of differentiation (osteogenic, adipogenic, and chondrogenic) was carried out in accordance with the manufacturer's instructions. Briefly, BMSCs were seeded in 6–well plates at a density of 1 × 10^5^ cells/well. When the confluence reached 80%, the osteogenic or adipogenic differentiation medium (Cyagen, CHINA) was substituted for osteogenic or adipogenic induction, respectively. After 14 days, osteogenesis was evaluated using alizarin red staining, and lipogenesis was evaluated using oil red O staining. For chondrogenic differentiation, suspension culture was employed, and BMSCs were cultured at a rate of 1 × 10^6^ cells/tube in chondrogenic differentiation medium (Cyagen, China) for 21 days. The precipitates were fixed, embedded, sectioned, and analyzed for chondrogenesis by Alcian Blue staining.

Human umbilical vein endothelial cells (HUVECs) and bone marrow‐derived macrophages (BMDMs) were purchased from Procell (Wuhan, China). HUVECs were cultured in complete endothelial cell medium (ECM; ScienCell). BMDMs were cultured in Dulbecco's modified Eagle's medium (DMEM) supplemented with 10% FBS and penicillin‐streptomycin (Gibco, USA; 1:100 dilution). Both cell types were maintained at 37°C in a humidified atmosphere containing 5% CO_2_

Additives, DUSP26 inhibitor (F1063‐0967) and MG132, were procured from MedChemExpress LTD. On the second day of differentiation, F1063–0967 and MG132 were added to the medium at concentrations of 20 and 10 µm, respectively. The medium was refreshed every 3 days.

### 5.2 RT‐qPCR

According to the manufacturer's instructions, total RNA was extracted using TRIzol reagent (Invitrogen, USA), and the first‐strand cDNA was synthesized using ReverTra Ace qPCR RT Kit(TOYOBO, Japan). RT‐qPCR was performed using SGExcel Universal SYBR qPCR Mix(Sangon, China) on a LineGene 9600 Plus system. After 3 min of pre‐denaturation at 95°C, the amplification process was carried out for 40 cycles at 95°C for 5 s and 60°C for 20 s. Then, it was melted at 95°C for 15 s and at 60°C for 1 min, and finally cooled at 60°C for 30 s. The primers used for RT‐qPCR are listed in Supplementary Table S1.

### 5.3 Western Blotting Assay

Cells were lysed for 30 min using radioimmunoprecipitation assay (RIPA) lysis buffer (Beyotime, China) containing a mixture of protease and phosphatase inhibitors (Beyotime, China), followed by protein quantification using bicinchoninic acid (BCA) reagent (Beyotime, China). The quantified protein lysates were separated on a 10% PAGE gel (Epizyme, China). Proteins were then transferred to a 0.22 µm polyvinylidene fluoride membrane (Millipore, USA), blocked with 5% skim milk for 2 h, and incubated overnight with primary antibodies at 4°C. Secondary antibodies were incubated for 1 h at room temperature, and detection was performed using enhanced  chemiluminescent reagent (Millipore, USA). Visualization was conducted using ChemiScope 6000 (Clinx, China). Western blotting antibody information is provided in Table S2.

### 5.4 Co‐IP

In brief, cells were lysed using RIPA lysis buffer containing protease inhibitors (Beyotime, China) for 30 min. The supernatant was then collected by centrifugation at 4°C and 13 000 rpm for 10 min. Antibodies were added according to the manufacturer's instructions and incubated at 4°C with thorough mixing overnight. On the following day, labeled magnetic beads were added and mixed at 4°C for 5 h. After three washes, loading buffer (Beyotime, China) was added and incubated at 100°C for 10 min prior to Western blotanalysis.

### 5.5 Cell Transfection

The shRNA and overexpression plasmids targeting DUSP26, YY1, PINK1, P300 and PMPCB genes, as well as the mutant plasmids for DUSP26, YY1, PINK1, P300 and PMPCB, were purchased from Ori‐Bio (China). The plasmids were transfected into 6‐well plate cells using Lipofectamine 3000 transfection reagent (Invitrogen, USA). Validation was performed using RT‐qPCR and Western blotting.

The RUNX2 promoter‐DUSP26 plasmid was synthesized by HANBIO (China Shanghai) Company.ShRNA information is provided in Table S4.

### 5.6 ALP Staining

BMSCs were incubated in osteogenic differentiation medium for 14 days. The cells were washed twice with phosphate‐buffered saline (PBS) and fixed in 4% paraformaldehyde (PFA) for 10 min at room temperature. Subsequently, ALP staining was performed using an ALP staining kit (Beyotime, China) at room temperature for 15 min, followed by two washes with double‐distilled water and imaging.

### 5.7 Alizarin Red S (ARS) Staining

BMSCs were incubated in osteogenic differentiation medium for 14 days. The cells were washed twice with PBS and fixed with 4% PFA at room temperature for 10 min, followed by two washes with double‐distilled water. Subsequently, the cells were stained with ARS solution (Solarbio, China) for 15 min, washed twice with double‐distilled water, and photographed.

### 5.8 ELISA

Markers, including VEGF, ANG2 (Abcam, USA), and SLIT3 (Coibo, China), were quantified from collected cell culture supernatants using commercially available ELISA kits, strictly following the manufacturer's guidelines. The absorbance at 450 nm was subsequently measured by a microplate reader (Thermo Fisher Scientific).

### 5.9 Preparation of CM

To prepare CM derived from BMSCs, 3 × 10^6 BMSCs with or without overexpression of DUSP26 were cultured in 10 cm dishes for 24 h. Subsequently, the medium was replaced with 15 mL DMEM/F‐12 without serum or antibiotics and incubated for an additional 48 h. Finally, the BMSC‐derived CM was harvested, centrifuged, and concentrated 20‐fold using a centrifugal tube (Millipore, USA). The concentrated CM was then quantified using the BCA protein assay and stored at −80°C for future use.

### 5.10 Evaluation of Cell Proliferation and Cell Viability

Cell proliferation was analyzed using the EdU detection kit (Beyotime, China). Equal volumes of culture medium were mixed with EdU working solution and incubated with HUVECs at 37°C for 2 h, followed by 4% polyformaldehyde fixation, sequential washing, and permeabilization. Click reaction solution was added to each sample and incubated for 30 min, after which Hoechst staining solution (Beyotime, China) was added. Observation was performed using a fluorescence microscope (Nikon, Japan). Cell viability was assessed by the CCK‐8 assay (MedChemExpress, USA). HUVEC treated with different conditions were incubated with CCK‐8 reagent at 37°C for 2 h, and absorbance measurements were taken at 450 nm for each group.

### 5.11 Transwell

Seed 5 × 10^3^ cells in the upper chamber, add DMEM/F‐12 or CMto the lower chamber, co‐culture for 24 h, then perform crystal violet staining and analyze by photographic counting.

### 5.12 Scratch Assay

Infect 1 × 10^3^ cells into 2‐well Ibidi chamber plates to form confluent monolayers. Subsequently, remove the plugs and wash the cells three times with PBS to remove suspended cells, followed by culture in serum‐free medium. Images were obtained using a microscope at 0, 6, and 12 h.

### 5.13 Tube formation Assay

The matrix gel (Corning, USA) was stored in a 4°C refrigerator the day prior to the experiment. HUVECs cells was performed in 6‐well plates for the experimental group. The culture plates, pipettes, and pipette tips were cooled in a −20°C refrigerator 2 ho before the experiment began. All procedures were conducted under ice conditions. A total of 200 µL of matrix gel was added to 24‐well plates and incubated at 37°C for coagulation. Both experimental and control groups of cells were digested with trypsin and centrifuged at 1000 rpm for 5 min to remove supernatants. Cells were resuspended in non‐double‐antibody DMEM/F‐12 or conditioned medium, and counts were performed using a cell counter plate. Cells were seeded at a density of 2 × 10^4 cells per well in 24‐well plates. Microscopic observations and photographs were recorded after 12 h.

### 5.14 In Vivo Experiment

All animal experiments were approved by the Medical Ethics Committee of the Second Affiliated Hospital of Harbin Medical University (YJSDW2024‐218).

Calcein double labeling assay: 8‐week‐old mice were intraperitoneally injected with calcarone (Sigma, USA, 1 mg/mL, 2% NaHCO3 solution) at doses of 20 mg/kg on days 0 and 4. On day 7, the mice were euthanized, followed by skeletal fixation, dehydration, and embedding. Samples were sectioned into 5 µm thin sections, and fluorescence‐labeled images were obtained using a microscope (BX51, Olympus). Performance analysis included BFR, MAR, and MS/BS.

Osteoporosis mouse model and treatment: Female mice aged 8 weeks were anesthetized and underwent bilateral ovariectomy via the midline dorsal approach to establish an estrogen‐deficient (OVX) osteoporosis mouse model. At 8 weeks postoperatively, the osteoporotic mice were randomly divided into two groups (*n* = 5) and treated with either a vector or AAV‐Sh‐DUSP26 (10 µL, viral titer: 2.62 × 10^1^ ^2^) via intramedullary injection into the femoral cavity. AAV‐Sh‐DUSP26 was purchased from Ori‐Bio (China). After 6 weeks of treatment, femoral bones from 22‐week‐old mice were collected for histological and micro‐CT analysis.

### 5.15 Micro‐CT

The right femur was obtained and fixed in 4% PFA for 2 days, followed by scanning with a MicroCT scanner (GE eXplore Locus SP micro‐CT, GE Healthcare, Barrington, IL, USA) at a working voltage of 90 kV, 88 µA, and a resolution of 9.0 µm/pixel. Three‐dimensional reconstruction and data processing were performed using reconstruction software based on the Feldkamp algorithm version 1.13.11.0. Regions of interest (ROIs) were defined for cortical and trabecular parameters. The trabecular ROI extended from 50 µm proximally to the distal growth plate end and extended more than 90 µm toward the diaphysis. Measured parameters included Tb.BMD, BV/TV, Tb.N, Tb.Th, and Tb.Sp,. The cortical bone ROI was defined as the 90 µm segment of the femoral diaphysis. Measured parameters included Cort.Th.

### 5.16 Histological Analysis

The left femur was fixed in 4% PFA for 2 days, decalcified with 10% ethylenediaminetetraacetic acid (EDTA) for about 8 weeks, and paraffin‐embedded. 2 µm sections were prepared for HE and multiplex immunofluorescence staining. H&E staining involved dewaxing, rehydration, hematoxylin staining, differentiation, eosin staining, dehydration, xylene clearing, and mounting for observation. Multiplex immunofluorescence experiments were performed as described in the kit instructions (AIFang Biological, China): rehydrated sections were blocked with 3% bovine serum albumin (BSA for 30 min, followed by overnight incubation with primary antibodies. The next day, fluorescently labeled secondary antibodies were incubated at room temperature for 30 min, and sections were sealed with DAPI‐containing fluorescent anti‐quenching mountant. Images were captured using a laser confocal microscope.

### 5.17 Immunofluorescence

The cell slide or frozen section was fixed at room temperature with 4% PFA for 10 min, followed by membrane disruption with 0.1% Triton X‐100 (Solarbio, China) for 2 min and blocking with 10% goat serum (Boster, China) for 30 min. The cells were then incubated with primary antibody at 4°C overnight under gentle shaking. According to the instructions of the Aifang Bio Dual‐Label Triple‐Color Multiplex Staining Kit, secondary antibody was incubated at room temperature in a dark room for 1 h, followed by dye re‐staining for 1 min. Finally, the cell slide was mounted with DAPI‐containing fluorescent anti‐quenching sealing agent and photographed.

### 5.18 Mitochondria, Nucleus, and Cytoplasmic Separation

Mitochondrial proteins were extracted using a mitochondrial extraction kit (Solarbio, China). Cells (1 × 10^7) were washed with pre‐cooled PBS, resuspended in solution A, homogenized, and centrifuged at 1000 g for 5 min at 4°C. The supernatant was centrifuged at 11 000 g for 15 min at 4°C to collect mitochondria. The pellet was treated with solution B and centrifuged at 11 000 g for 10 min at 4°C. Mitochondrial proteins were obtained by resuspending the pellet in lysis buffer with protease inhibitors and shaking vigorously.

For nuclear and cytoplasmic protein extraction using a kit (Epizyme, China), collect 1 × 10^7 cells and wash twice with pre‐cooled PBS. Add 200 µL Reagent A, mix, incubate at ice 10 min. Add 10 µL Reagent B, vortex 5 sec, incubate at ice 1 min. Vortex 5 sec, incubate at ice 2 min. Centrifuge at 4°C, 1600 g for 10 min; collect supernatant for cytoplasmic proteins. Add 100 µL Reagent C to pellet, mix, vortex 10 sec, incubate at ice for 10 min, centrifuge at 4°C, 11 000 g for 10 min; collect supernatant for nuclear proteins.

### 5.19 JC‐1

The procedure was carried out in accordance with the instructions of the Mitochondrial Membrane Potential Detection (JC – 1) Kit (Solarbio, China). Initially, 50 µL of JC – 1 (200×) was diluted with 8 mL of ultrapure water. Subsequently, the mixture was incubated in a 37°C water bath for 20 min. After removal, the mixture was shaken to guarantee the complete dissolution of the precipitate. Next, 2 mL of JC – 1 staining buffer (5×) was added and thoroughly mixed to obtain the JC – 1 working solution. Hoechst was added to the JC – 1 staining working solution at a ratio of 1:100 and vigorously mixed. The original culture medium was removed, and the mixed JC – 1 staining working solution was transferred. The sample was incubated in a 37°C incubator for 20 min. After incubation, the supernatant was aspirated, the sample was washed three times with PBS, and the sample was observed and photographed under a laser confocal microscope.

### 5.20 MitoSOX

Following the instructions of the MitoSOX reagent (Invitrogen, USA), first dissolve the contents of the reagent bottle in 13 µL of anhydrous dimethyl sulfoxide to prepare a 5 mm solution. Then add 5 µL of the 5 mm reagent solution to 50 mL of complete medium to prepare a 500 nm MitoSOX working solution. Remove the original medium and add the mixed MitoSOX staining working solution. Incubate in a 37°C incubator for 20 min. After incubation, aspirate the supernatant, wash with PBS three times, and observe and photograph under a laser confocal microscope.

### 5.21 TEM

Cells were fixed in a solution containing 2.5% glutaraldehyde. Secondary fixation was performed using a 2% aqueous osmium tetroxide solution, followed by dehydration through a concentration gradient of ethanol (30%–100%) and epoxy resin infiltration. The sample was cured at 60°C for 24 h. The resin block was cut into 60–80 nm sections using a supermicrotome and transferred onto a 150‐well copper grid. Samples were immersed in a 2% uranium acetate alcohol solution, light‐exposed for 8 min to avoid staining, then rinsed three times with 70% ethanol and three times with ultrapure water. Subsequently, they were soaked in a 2.6% lead citrate solution for 8 min under CO_2_‐free conditions, followed by three additional rinses with ultrapure water. After drying on filter paper, the copper grid was placed on a grid plate and dried overnight at room temperature. The grids were examined under a TEM, and images were captured using a slow‐scan camera with iTEM software.

### 5.22 Double‐Labeled Lentivirus mRFP‐GFP‐LC3

According to the manufacturer's instructions (HANBIO, China), add 1/2 volume of diluted adenovirus culture medium to the cells at infection multiplicity of infection (MOI) of 0, 20, 40, 60, 80, or 100. Infect the cells at 37°C for 4 h, then supplement the medium to the normal volume. The infection efficiency was observed 24 h post‐infection to determine the optimal MOI. Under the optimal MOI transfection conditions, osteogenic induction was observed and photographed under laser confocal microscopy 7 days later.

### 5.23 Protein Stability Experiment

Cycloheximide (CHX, Sigma, USA) was added to the cell culture medium at concentrations of 0, 10, 20, 50, 100, and 200 µg/mL, respectively. Cells were cultured at 37°C for 6 and 12 h, followed by determination of the optimal concentration at 50 µg/mL. At this optimal concentration, cells were treated and cultured for 6 and 12 h, after which detection was performed via Western blot.

### 5.24 ChIP

The ChIP assay was performed using a SimpleChIP Plus Enzymatic Chromatin IP Kit (CST, USA). Briefly, 1 × 10^7^ BMSC, after fixation, were resuspended in ChIP buffer containing 1 × Protease Inhibitor Cocktail (CST). Then, micrococcal nuclease was used to shear chromatin DNA into fragments ranging between 150 and 900 bp. The DNA fragments were immunoprecipitated with 10 µL precleared protein A/G agarose beads overnight at 4°C with anti‐YY1 (Abclonal, China) or anti‐IgG (Proteintech, China) antibodies. YY1 occupancy on the DUSP26 promoter was examined using quantitative PCR analysis. The primers used for ChIP‐qPCR are listed in Supplementary Table S1.

### 5.25 Luciferase Activity Assays

PCR amplification of the target gene promoter from mouse genomic DNA, followed by insertion into pGL3‐Basic, generated the firefly luciferase reporter plasmid. Transfection introduced the reporter plasmid into BMSCs at approximately 75% confluence. After 48 h, a dual‐luciferase reporter assay kit (Vazyme, Cat. No. DL101‐01) measured firefly and Renilla luciferase activities. Renilla luciferase activity served as the internal control for normalization of firefly luciferase reporter activity.

### 5.26 FAIRE‐qPCR

Cells were cultured to approximately 95% confluence and fixed with 1% paraformaldehyde (PFA) for 15 min at room temperature. Addition of 0.125 m glycine quenched the crosslinking reaction. The cells were then washed with ice‐cold PBS supplemented with 1 mm phenylmethylsulfonyl fluoride (PMSF), collected, and lysed in SDS lysis buffer (1% SDS, 10 mm EDTA, 50 mm Tris‐HCl, pH 8.1, 1 mm PMSF, and 1 mm protease inhibitor cocktail) for 10 min on ice. The lysate was divided into two equal aliquots, including one designated for control DNA. Following the addition of 10 µL proteinase K (20 mg/mL), the sample was incubated at 37°C for 4 h. DNA from the non‐reverse‐crosslinked and reverse‐crosslinked samples was then purified by phenol‐ chloroform extraction. The primers used for FAIRE‐PCR are listed in Supplementary Table S1.

### 5.27 mt‐Keima Flow Cytometry

BMSCs were transfected with a mitochondria‐targeted Keima reporter and subjected to the indicated gene perturbations. Neutral and acidic mt‐Keima signals were acquired by flow cytometry using 405 and 561‐nm excitation, respectively. Mitophagy was quantified as the proportion of cells with acidic mt‐Keima fluorescence and the acidic‐to‐neutral fluorescence ratio.

### 5.28 Recombinant DUSP26 and pNPP Phosphatase Assay

FLAG‐tagged DUSP26‐WT and DUSP26‐C152S were purchased from BioBasic at > 95% purity and assessed by SDS‐PAGE and Coomassie Brilliant Blue staining. Phosphatase activity was measured using the SensoLyte pNPP Protein Phosphatase Assay Kit (AS‐71105; AnaSpec). Each well contained 50 µL of protein sample and 50 µL of pNPP working solution prepared with the supplied substrate, assay buffer, and dithiothreitol (DTT). For the time course, 15 µg of protein was incubated at 37°C for 0–60 min. For the dose assay, 0, 2.5, 5, or 7.5 µg of protein was incubated at 37°C for 30 min. Reactions were terminated with 50 µL of stop solution, shaken for 1 min, and read at 405 nm. No‐protein blanks were processed in parallel and subtracted from sample readings.

### 5.29 In Vitro Dephosphorylation

PMPCB was immunopurified from BMSCs lysed in NP‐40 lysis buffer without phosphatase inhibitors and used as the substrate in a cell‐free dephosphorylation assay. After purification, the PMPCB‐bound beads were washed three times with sterile PBS. Then the PMPCB was eluted. Purified PMPCB (1 µg) was incubated with FLAG‐DUSP26‐WT or FLAG‐DUSP26‐C152S (BioBasic; purity > 95%; 0.25 µg) at 37°C for 30 min in an approximately 300 µL reaction containing 50 mm 4‐(2‐hydroxyethyl)‐1‐piperazineethanesulfonic acid (HEPES), 100 mm NaCl, 1 mm MnCl_2_, 2 mm DTT, 0.1 mm ethylene glycol‐bis(β‐aminoethyl ether)‐N,N,N′,N′‐tetraacetic acid, and 0.025% Tween 20. A PMPCB‐containing reaction without DUSP26 served as the control. Reactions were terminated by adding loading buffer (Beyotime, China) and heating at 100°C for 10 min. Reaction products were analyzed by Western blotting using pan‐phosphotyrosine and pan‐phosphoserine/threonine antibodies to assess PMPCB phosphorylation.

### 5.30 LC–MS/MS and PMPCB Sequence Comparison

Endogenous PMPCB was immunoprecipitated from BMSCs expressing DUSP26‐WT or DUSP26‐C152S. Samples were transferred to 10‐kDa filters, washed five times with PBS, denatured in 30 µL of 8 m urea/50 mm ammonium bicarbonate, reduced with 10 mm DTT at 55°C for 30 min, and alkylated with 15 mm iodoacetamide for 30 min in the dark. After urea dilution to < 1 m, proteins were digested with 0.4 µg of trypsin overnight at 37°C. Peptides were collected at 12 000 rpm for 5 min, desalted on C18 columns, and eluted twice with 40 µL of 50% acetonitrile/0.1% formic acid.

Peptides were analyzed by nano‐LC coupled to a Q Exactive Plus mass spectrometer. Separation used a C18 trap column (100 µm × 20 mm) and an analytical column (75 µm × 250 mm) at 300 nL/min. Solvent B was 0.1% formic acid in acetonitrile; the gradient increased from 5% to 38% B over 50 min, reached 95% B at 52 min, and was held to 60 min. Full MS scans (m/z 300–1500) were followed by MS/MS of the 20 most intense precursors using HCD at a normalized collision energy of 28 and 25‐s dynamic exclusion. MS1 settings were 70 000 resolution, 3 × 10^6^ AGC, and 100‐ms maximum injection time; MS2 settings were 17,500 resolution, 1 × 10^5^ AGC, and 50‐ms maximum injection time.

PMPCB sequences from human, mouse, rat, Callithrix jacchus, Macaca mulatta, Chlorocebus sabaeus, and Mesocricetus auratus were aligned using human PMPCB (UniProt O75439) as the reference to assess conservation of the residue corresponding to Y141.

### 5.31 Size‐Exclusion Chromatography of MPP Complexes

BMSCs were transfected with plasmids encoding C‐terminally 3×FLAG‐tagged PMPCB‐WT or PMPCB‐Y141F. After protein expression, mitochondria were isolated from the transfected cells, and PMPCB‐containing protein complexes were purified from the mitochondrial lysates by anti‐FLAG affinity purification. PMPCA co‐purified with FLAG‐tagged PMPCB to form the MPP complex. The FLAG‐affinity eluates were subsequently subjected to size‐exclusion chromatography on a Superdex 200 Increase 10/300 GL column (Cytiva) connected to an ÄKTA pure 25 chromatography system. Chromatography was performed at 4°C and a flow rate of 0.5 mL/min using 20 mm HEPES‐NaOH (pH 7.5), 100 mm NaCl, and 1 mm tris(2‐carboxyethyl)phosphine (TCEP) as the mobile phase. PMPCB‐WT and PMPCB‐Y141F preparations were processed under identical conditions. Fractions corresponding to the principal peak, with an apparent molecular mass of approximately 105–110 kDa, and the secondary peak, with an apparent molecular mass of approximately 50–55 kDa, were collected separately. The principal peak was assigned to the PMPCA–PMPCB heterodimer, whereas the secondary peak corresponded predominantly to excess PMPCB‐WT or PMPCB‐Y141F monomer. The collected fractions were analyzed by SDS‐PAGE and Coomassie Brilliant Blue staining. Fractions containing the purified MPP heterodimer were pooled for the cell‐free PINK1 processing assay, whereas PMPCB‐containing monomer fractions were used for the indicated biochemical analyses.

### 5.32 Surface Plasmon Resonance and Binding Analysis

Surface plasmon resonance was performed at 25°C using a Biacore X100 with a Series S CM5 sensor chip. Recombinant mouse PMPCA (BioBasic; purity > 95%) was immobilized on flow cell 2 by amine coupling (∼ 18,321 RU), and flow cell 1 served as the reference. PBS‐P+ containing 20 mm phosphate, 137 mm NaCl, 2.7 mm KCl, and 0.05% Surfactant P20 (pH 7.35) was used as the running and sample‐dilution buffer. In‐house SEC‐purified PMPCB‐WT or PMPCB‐Y141F (0, 6.25, 12.5, 25, 50, 100, and 200 nm) was injected at 30 µL/min for 60 s, followed by 200 s of dissociation and a 50% dimethyl sulfoxide wash.

Reference‐subtracted sensorgrams (Fc2 − Fc1) were baseline‐corrected using the mean response at 20–50 s, and the matched zero‐concentration trace was subtracted. Three technical measurement series were fitted globally to a 1:1 binding model to obtain ka, kd, and KD (kd/ka). Apparent steady‐state KD was obtained by fitting the response averaged over the final 10 s of the association phase to a one‐site binding model.

### 5.33 Cell‐Free PINK1 Processing Assays

SEC fractions containing WT‐MPP or Y141F‐MPP heterodimers were pooled and concentrated, and protein purity was assessed by SDS‐PAGE and Coomassie Brilliant Blue staining. Untagged full‐length mouse PINK1 (BioBasic; purity > 95%; 10 µm) was incubated with WT‐MPP or Y141F‐MPP (0.5 µm) in 10 µL of 20 mm HEPES‐KOH (pH 7.5) and 1 mm TCEP at 37°C for 60 min. PMPCA alone and PMPCB alone served as controls. Reactions were terminated with an equal volume of 2× SDS sample buffer and heated at 100°C for 10 min. PINK1 processing was assessed by Western blotting using anti‐PINK1 antibody.

### 5.34 Exosome Preparation, Plasmid Loading, and Characterization

Extracellular vesicles derived from C57BL/6 mouse bone marrow mesenchymal stem cells (OriCell, MUBMX‐06001; Cyagen Biosciences) were purchased and used as exosomes. For each loading reaction, 10 µg of exosome protein and 5 µg of RUNX2 promoter‐driven DUSP26 plasmid were mixed in 50 µL of electroporation buffer containing 1.15 mm potassium phosphate (pH 7.2), 25 mm KCl, and 21% OptiPrep. Samples were electroporated at 400 V and 125 µF with two pulses using a Gene Pulser Xcell system; mock‐electroporated exosomes without plasmid served as controls. After recovery with 1 mm EDTA for 15 min, free or surface‐associated DNA was removed by 300‐kDa ultrafiltration and deoxyribonuclease I (DNase I) treatment (1.5 Kunitz units/µL, 20 min). DNase I was stopped with 20 mm EDTA, and the vesicles were washed with sterile PBS.

Vesicle morphology was examined by TEM after 2% uranyl acetate staining. Particle size and zeta potential were measured using a Flow NanoAnalyzer and Zetasizer Nano ZS, respectively, with four independent preparations per group.

### 5.35 Plasmid Loading Measurement and Expression Assays

Plasmid DNA was measured using the Quant‐iT PicoGreen dsDNA (Invitrogen, USA)assay with a 0–300 ng/mL standard curve and fluorescence detection at 480/520 nm. Loading efficiency was calculated as plasmid DNA associated with the purified loaded‐vesicle preparation divided by the input plasmid DNA × 100%. BMSCs, HUVECs, and BMDMs were treated with RUNX2‐DUSP26‐EXO, and DUSP26 mRNA was measured by RT‐qPCR. EXO, RUNX2‐DUSP26‐EXO, and CMV‐DUSP26‐EXO were also compared in BMSCs.

### 5.36 In Vivo Exosome Distribution and Transgene Expression

Exosomes and PKH67 working solution were prepared separately in kit diluent, mixed at equal volumes, and incubated for 3 min at room temperature in the dark. Labeling was quenched with an equal volume of medium containing 10% exosome‐depleted FBS. Free dye was removed by PBS washing and vesicle repurification. Unlabeled exosomes and a dye‐only preparation processed through the same cleanup steps served as controls.

PKH67‐labeled exosomes were administered by tail‐vein injection. Femora and major organs were collected at the designated time. Femoral frozen sections were examined for PKH67 fluorescence and anti‐FLAG immunofluorescence using a Zeiss LSM 900 with identical acquisition settings across groups. Bone marrow and dissociated organs were passed through 70‐µm strainers, subjected to erythrocyte lysis when required, and resuspended in PBS containing 2% FBS.

For viability analysis, up to 1 × 10^6^ cells were incubated in 1 mL of 1 × 7‐aminoactinomycin D solution (20× stock diluted 1:20) for 5 min at 37°C in the dark and analyzed immediately. A matched aliquot for intracellular FLAG detection was surface‐stained, fixed, and permeabilized, then incubated with anti‐FLAG antibody (Proteintech, 66008‐4‐Ig; 0.25 µg/10^6^ cells in 100 µL) for 20 min at room temperature and a PE‐conjugated secondary antibody. LepR, CD31, and F4/80 surface staining was performed after Fc‐receptor blockade for 30 min at 4°C in the dark. Cells were gated by FSC/SSC, singlets, viability, and lineage markers; PKH67 and FLAG signals were collected in the FITC and PE channels, respectively. Unstained, single‐color, fluorescence‐minus‐one, isotype, unlabeled‐exosome, and dye‐only controls were included. Flow cytometry antibodies and isotype controls are listed in Table S3.

### 5.37 Cellular Uptake Assay

For the cell uptake study, red fluorescent PKH‐26 (Beyotime,C2071) was used to label exosomes, while green fluorescent PKH‐67 (Beyotime, C2073) was employed to label the cell membrane. DAPI staining (Beyotime,C1006) was used for visualizing cell nuclei. BMSCs were treated overnight with exosomes labeled with the fluorescent dye PKH‐26. Following fixation, the internalization of labeled exosomes by BMSC was visualized via laser scanning confocal microscopy.

### 5.38 Statistical Analysis

All data were expressed as mean ± SD. Statistical analysis and presentation were performed using ImageJ and GraphPad 10.0. Univariate analysis of variance and Bonferroni multiple comparison test were employed for statistical analysis. A P‐value < 0.05 was considered statistically significant, with ^*^ *p* < 0.05, ^**^ *p* < 0.01, and ^***^ *p* < 0.001 indicating significant differences.

## Author Contributions

**Yunpeng Lv**: writing – review and editing, writing – original draft, methodology, validation, supervision, data curation, software, formal analysis, conceptualization, investigation, visualization, project administration. **Feng Wu**: validation, formal analysis. **Xiongjie Liang**: validation, formal analysis. **Chong Liu**: validation, formal analysis. **Wenhui Guo**: methodology, software. **Kejian Zhang**: methodology, software. **Jiawen Niu**: conceptualization. **Wenjie Jiang**: conceptualization, investigation. **Hongjian Xu**: conceptualization, investigation. **Anlong Jiang**: investigation, conceptualization. **Yang Zheng**: conceptualization, investigation. **Enming Yu**: conceptualization, investigation. **Xintao Wang**: conceptualization, investigation, resources. **Jinglong Yan**: writing – review and editing, funding acquisition, visualization, project administration, resources, supervision, data curation, software, formal analysis, validation, methodology, conceptualization, investigation. **Jingjun Xia**: methodology, software, data curation, supervision, formal analysis, investigation, funding acquisition, visualization, project administration, resources. **Chengchao Song**: writing – review and editing, funding acquisition, visualization, project administration, resources, supervision, data curation, software, formal analysis, validation, methodology, conceptualization, investigation.

## Funding

This work was supported by the National Natural Science Foundation of China, Grant/Award Number: 82402860; Natural Science Foundation of Heilongjiang Province, Grant/Award Number: LH2023H040; National Natural Science Foundation of China, Grant/Award Number: U23A20412; Heilongjiang Postdoctoral Financial Assistance, Grant/Award Number: LBH‐Z22228; 2023 Heilongjiang Provincial Universities Basic Scientific Research Business Expenses; Scientific Research Project, Grant/Award Number: 2023‐KYYWF‐0190; New Era Longjiang Outstanding Master's and Doctoral Dissertations Project Funding, Grant/Award Number: LJYXL2022‐070; Chun‐Yan Innovation Team Program of the Heilongjiang Province, Grant/Award Number: CYCX24016.

## Conflicts of Interest

The authors declare no conflicts of interest.

## Supporting information




**Supporting File**: advs78514‐sup‐0001‐SuppMat.docx.

## Data Availability

The data that support the findings of this study are available from the corresponding author upon reasonable request.
