# Supplementary material for: DUSP26 Regulated by YY1 Lactylation Couples Osteogenesis and Angiogenesis by Dephosphorylating PMPCB at Y141 and Activating Mitophagy in BMSCs
Source: Adv Sci (Weinh). 2026 Oct 28:e78514. Online ahead of print. doi: 10.1002/advs.78514 (PMC13715922; doi:10.1002/advs.78514)
Supplement: Supplementary file 1 [file ADVS-9999-e78514-s001.docx]

Supporting Information

**DUSP26 regulated by YY1 lactylation couples osteogenesis and angiogenesis by dephosphorylating PMPCB at Y141 and activating mitophagy in BMSCs**

*Yunpeng Lv^1^. Feng Wu^1^. Xiongjie Liang^1^. Chong Liu^2^. Wenhui Guo^1^. Kejian Zhang^1^. Jiawen Niu^1^. Wenjie Jiang^1^. Hongjian Xu^1^*. Anlong Jiang*^1^*. *Yang Zheng^3^*. *Enming Yu^1^. Xintao Wang^1^* *Jinglong Yan^1,^*, Jingjun Xia^1,^*,* Chengchao Song*^1,^**

**This document includes:**

Figures S1 to S6

Tables S1 to S4

**Supplementary Figures**

**Figure S1. DUSP26 influences the secretion of angiogenic factors.**(A-B) ELISA measurements of VEGF, ANG2, and SLIT3 in supernatants from BMSCs with DUSP26 knockdown (A) or overexpression (B). (n = 12).

**Figure S2. Effect of YY1 lactylation on DUSP26.** (A) FAIRE assays were performed 48 h after BMSCs were transfected with scrambled control shRNA or sh-YY1. The recovered DNA was analyzed by qPCR to quantify enrichment of the DUSP26 promoter region (n = 3). (B) RT-qPCR analysis was performed to evaluate the effect of YY1 on DUSP26 mRNA expression in the absence of osteogenic induction (n = 12). (C) IP analysis was used to detect changes in YY1 lactylation after induction (n = 3). (D) IP (n = 3) was employed to detect the lactylation levels of K183R and the impact of YY1 lactylation on DUSP26 mRNA expression. (E,F) ALP and ARS staining were used to assess the effects of p300 on osteogenic differentiation in BMSCs transfected with YY1 WT or YY1 K183R. (n=3) （G,H）ELISA was used to assess the effects of p300 on the secretion of proangiogenic factors in BMSCs transfected with YY1 WT or YY1 K183R.(n = 12)** *p* < 0.01, *** *p* < 0.001,**** *p* < 0.0001 , ns, not significant.

**Figure S3. Effect of DUSP26 on mitochondrial autophagy.** (A) mRFP-GFP-LC3 analysis of autophagy-associated fluorescent puncta following DUSP26 knockdown. (n = 3).Scale bar = 10μm. Quantitative analysis of mRFP-GFP-LC3 (n = 3). (B) ELISA analysis of the effects of DUSP26 overexpression on the secretion of proangiogenic factors by PINK1-knockdown BMSCs. (C) Immunofluorescence analysis of PINK1 staining in LepR-positive cells in distal femoral bone (n = 5). Scale bar = 100μm. ** p < 0.01, *** p < 0.001,**** p < 0.0001 , ns, not significant.

**Figure S4. DUSP26 mediates osteogenesis and angiogenesis through the dephosphorylation of PMPCB protein.** (A) Western blotting was used to evaluate the effect of DUSP26 on the protein stability of PMPCB (n = 3). (B) IF analysis was used to detect the effect of DUSP26 on the cellular localization of PMPCB protein. Scale bar = 50μm. (C) Flow cytometry assessed the effects of DUSP26 WT and DUSP26 C152S on mitophagy in PMPCB-overexpressing BMSCs. (D-E) ALP and ARS staining assessed osteogenic differentiation, and ELISA measured proangiogenic factor secretion in PMPCB-overexpressing BMSCs expressing DUSP26 WT or DUSP26 C152S. (F) Western blotting assessed the effect of DUSP26 knockdown on PINK1 processing in BMSCs expressing PMPCB-WT or PMPCB-Y141F. (G-I) mt-Keima flow cytometry, ALP and ARS staining, and ELISA assessed the effects of DUSP26 knockdown on mitophagy, osteogenic differentiation, and proangiogenic factor secretion in BMSCs transfected with PMPCB-WT or PMPCB-Y141F. ** p < 0.01, *** p < 0.001,**** p < 0.0001 , ns, not significant.

**Figure S5. Characterization and Biosafety of Engineered Exosomes.** (A) A standard curve was generated using serial dilutions of plasmid DNA (0–300 ng/mL), with fluorescence measured at excitation/emission wavelengths of 480/520 nm. (B) RT-qPCR analysis of DUSP26 mRNA levels in different cell types treated with RUNX2-DUSP26-EXO. (C) Comparison of DUSP26 mRNA levels in BMSCs treated with control or engineered exosome preparations. (D) Flow cytometry assessed engineered EXO uptake and FLAG-DUSP26 expression in cells isolated from various mouse organs. (E) Flow cytometry of bone marrow single-cell suspensions assessed FLAG-DUSP26 expression in osteogenic-lineage cells, immune cells, and endothelial cells. (F) HE staining was employed to evaluate the biocompatibility of RUNX2-DUSP26-EXO. Scale bar = 100μm.

**Figure S6. Safety assessment and additional efficacy analyses of engineered exosomes.** (A) Complete blood count and serum biochemistry analyses were performed to evaluate the biocompatibility of RUNX2-DUSP26-EXO (n = 5).(B) Body weight of mice after treatment with RUNX2-DUSP26-EXO (n = 5). (C-D) Quantification of OCN fluorescence intensity (C) and type H vessel area (D) (n = 5).

**Supplementary Tables**

**Table S1:Primers**

| Primers for qRT-PCR | Primer sequence (5′ to 3′) |
| --- | --- |
| Mouse DUSP26 Forward | CGACTCTCCAGCCTTTGATATG |
| Mouse DUSP26 Reverse | CACAGTGCACCAGGATCTTC |
| Human DUSP26 Forward | AACCTGTCTTGGGCAGAAAC |
| Human DUSP26 Reverse | ATGGGTTCAGTTGCCAGGTA |
| Mouse RUNX2-Forward | GCCACTTACCACAGAGCTATT |
| Mouse RUNX2-Reverse | GAGGCGATCAGAGAACAAACT |
| Mouse ALP-Forward | GGCCTGGATCTCATCAGTATTT |
| Mouse ALP-Reverse | AGAGACCTAAGAGGTAGTCCAC |
| Mouse ACTIN β-Forward | GAGGTATCCTGACCCTGAAGTA |
| Mouse ACTIN β-Reverse | CACACGCAGCTCATTGTAGA |
| Human ACTIN β-Forward | AGGCACCAGGGCGTGAT |
| Human ACTIN β-Reverse | GCCCACATAGGAATCCTTCTGAC |
| Mouse YY1 Forward | GAAGATGATGCTCCAAGAAC |
| Mouse YY1 Reverse | TCTCCAGTATGAACCAGTTG |
| Mouse PMPCB Forward | GATTTGCCCAGAGCTGTAGAA |
| Mouse PMPCB Reverse | TCCTGCATCTCTCTCAGGATTA |
| Primers of ChIP-qPCR | Primer sequence (5′ to 3′) |
| Dusp26 ChIP Forward | CTGCTGACCTGGACTTAGCC |
| Dusp26 ChIP Reverse | TCATGAATTGCAGACGGCCT |
| Primers for FAIRE-qPCR | Primer sequence (5′ to 3′) |
| Dusp26 FAIRE Forward | GCTTCGATGTGCGTGTCATG |
| Dusp26 FAIRE Reverse | GTCGCAAGGGTCTCAGATCC |
| ACTIN β FAIRE Forward | AAAGGCAACTTTCGGAACGG |
| ACTIN β FAIRE Reverse | TTCCTCAATCTCGCTCTCGC |

**Table S2: Antibodies for Western blotting**

| Antibodies | Product code | Dilution | Supplier |
| --- | --- | --- | --- |
| DUSP26 Polyclone antibody | GTX109283 | 1:1000 | GeneTex |
| ALP Polyclone antibody | DF6225 | 1:1000 | Affinity |
| RUNX2 Polyclone antibody | 20700-1-AP | 1:1000 | Proteintech |
| YY1 Polyclone antibody | A19569 | 1:1000 | ABclonal |
| PanKla Polyclone antibody | PTM-140RM | 1:1000 | PTM |
| LaminB Polyclone antibody | 12987-1-AP | 1:5000 | Proteintech |
| COXIV Polyclone antibody | 11242-1-AP | 1:5000 | Proteintech |
| PINK1 Polyclone antibody | 23274-1-AP | 1:1000 | Proteintech |
| Parkin Polyclone antibody | 14060-1-AP | 1:1000 | Proteintech |
| P62 Polyclone antibody | AF5384 | 1:1000 | Affinity |
| LC3B Polyclone antibody | CTX127375 | 1:1000 | GeneTex |
| TOM20 Polyclone antibody | 11802-1-AP | 1:5000 | Proteintech |
| PMPCB Polyclone antibody | 16064-1-AP | 1:1000 | Proteintech |
| pSer/Thr Polyclone antibody | AP1475 | 1:1000 | ABclonal |
| pTyr Monoclonal antibody | 86398-1-RR | 1:1000 | Proteintech |
| P-Ser/Thr/Tyr Rabbit pAb | AB_2936786 | 1:1000 | Abmart |
| VEGF Polyclone antibody | 30980-1-AP | 1:1000 | Proteintech |
| Slit3 Polyclone antibody | DF9909 | 1:1000 | Affinity |
| ANGII Polyclone antibody | 24613-1-AP | 1:1000 | Proteintech |
| p300 Polyclonal antibody | 20695-1-AP | 1:1000 | Proteintech |
| PMPCA Monoclonal antibody | A24606 | 1:1000 | ABclonal |
| Beclin1 Polyclonal antibody | 11306-1-AP | 1:10000 | Proteintech |
| CD9 Monoclonal antibody | 98327 | 1:1000 | CST |
| CD63 Monoclonal antibody | ab217345 | 1:1000 | Abcam |
| TSG101 Polyclonal antibody | 28283-1-AP | 1:10000 | Proteintech |
| Calnexin Polyclonal antibody | 10427-2-AP | 1:50000 | Proteintech |
| Specific secondary antibody used to avoid the influence of (H+L) | M21008 | 1:5000 | Abmart |
| GAPDH Monoclonal antibody | 60004-1-Ig | 1:50,000 | Proteintech |
| OCN Polyclonal antibody | ab93876 | 1:100 (IF/IHC) | Abcam |
| LepR Polyclonal antibody | AF497 | 10 μg/mL (IF/IHC) | R&D Systems |
| CD31 Monoclonal antibody | ab182981 | 1:500 (IF/IHC) | Abcam |

**Table S3: Antibodies for flow cytometry**

| Antibodies | Product code | Application and dilution | Supplier |
| --- | --- | --- | --- |
| CD31 Monoclonal antibody | ab182981 | 0.25 μg/test in 100 μL (FC) | Abcam |
| FLAG-tag Monoclonal antibody | 66008-4-Ig | 0.25 μg/10⁶ cells in 100 μL (FC) | Proteintech |
| APC-conjugated LepR antibody | FAB497A | 0.25 μg/test in 100 μL (FC) | R&D Systems |
| Alexa Fluor 700-conjugated F4/80 antibody | 56-4801-82 | 0.25 μg/test in 100 μL (FC) | Invitrogen/eBioscience |

**Table S4: shRNA target sequences**

| shRNA | Target sequence (5′ to 3′) |
| --- | --- |
| shDUSP26 | GCCTGTAACCATGCTGACGAA |
| shYY1 | CGACGGTTGTAATAAGAAGTT |
| shp300 | CCCTGGATTAAGTTTGATAAA |
| shPINK1 | GGAGCAGTTACTTACAGAAGA |
| shPMPCB | GGGATTTACACGAAGGCTT |
| shNC | GGTGGTATGGAAGCTAATA |
